# Advanced Approaches to Breast Cancer Classification and Diagnosis

**DOI:** 10.3389/fphar.2020.632079

**Published:** 2021-02-26

**Authors:** M. Zubair, S. Wang, N. Ali

**Affiliations:** ^1^ Department of Biology, University of Arkansas at Little Rock, Little Rock, AR, United States; ^2^ Department of Chemistry, University of Arkansas at Little Rock, Little Rock, AR, United States

**Keywords:** breast cancer, heterogeneity, novel biomarkers, early diagnosis, multigene assays, biosensors

## Abstract

The International Agency for Research on Cancer (IARC) has recently reported a 66% increase in the global number of cancer deaths since 1960. In the US alone, about one in eight women is expected to develop invasive breast cancer(s) (breast cancer) at some point in their lifetime. Traditionally, a BC diagnosis includes mammography, ultrasound, and some high-end molecular bioimaging. Unfortunately, these techniques detect BC at a later stage. So early and advanced molecular diagnostic tools are still in demand. In the past decade, various histological and immuno-molecular studies have demonstrated that BC is highly heterogeneous in nature. Its growth pattern, cytological features, and expression of key biomarkers in BC cells including hormonal receptor markers can be utilized to develop advanced diagnostic and therapeutic tools. A cancer cell's progression to malignancy exhibits various vital biomarkers, many of which are still underrepresented in BC diagnosis and treatment. Advances in genetics have also enabled the development of multigene assays to detect genetic heterogeneity in BC. However, thus far, the FDA has approved only four such biomarkers—cancer antigens (CA); CA 15-3, CA 27-29, Human epidermal growth factor receptor 2 (HER2), and circulating tumor cells (CTC) in assessing BC in body fluids. An adequately structured portable-biosensor with its non-invasive and inexpensive point-of-care analysis can quickly detect such biomarkers without significantly compromising its specificity and selectivity. Such advanced techniques are likely to discriminate between BC and a healthy patient by accurately measuring the cell shape, structure, depth, intracellular and extracellular environment, and lipid membrane compositions. Presently, BC treatments include surgery and systemic chemo- and targeted radiation therapy. A biopsied sample is then subjected to various multigene assays to predict the heterogeneity and recurrence score, thus guiding a specific treatment by providing complete information on the BC subtype involved. Thus far, we have seven prognostic multigene signature tests for BC providing a risk profile that can avoid unnecessary treatments in low-risk patients. Many comparative studies on multigene analysis projected the importance of integrating clinicopathological information with genomic-imprint analysis. Current cohort studies such as MINDACT, TAILORx, Trans-aTTOM, and many more, are likely to provide positive impact on long-term patient outcome. This review offers consolidated information on currently available BC diagnosis and treatment options. It further describes advanced biomarkers for the development of state-of-the-art early screening and diagnostic technologies.

## Introduction

**FIGURE 1:**
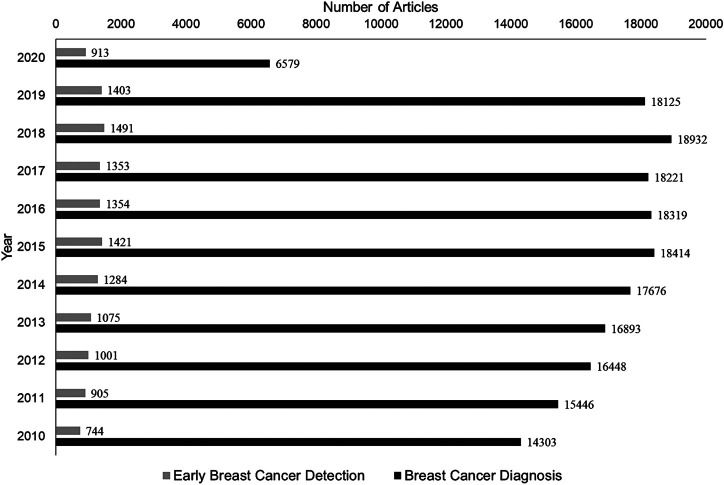
A comparative analysis of the number of articles published in the last decade, limiting the search to keywords: “Breast Cancer Diagnosis” and “Early Breast Cancer Detection.” NCBI database was searched as on Nov 1, 2020, to acquire the respective number of publications shown.

Cancer cells are misbehaving normal cells that are beyond the paradigm of life and death. Some researchers consider their self-sufficiency and self-management as an evolutionary process in the cell division. Like an organism that evolves through a process of natural selection and mutation, cancer cells also progress through selective transformation to malignancy (Casás-Selves and DeGregori, 2011). The current edition of the International Agency for Research on Cancer (IARC) reports a 66% increase in the global number of cancer deaths since 1960. Currently, breast cancer(s) (BC) is the second most common cancer worldwide, after lung cancer. Accordingly, in the US alone, about one in eight women is expected to develop invasive BC at some point in their lifetime. Considering the number of research articles published on BC diagnosis and treatments, research in its early detection is still lagging significantly (Figure 1). In this review, we aim at providing consolidated information on recent advancements in BC diagnosis and therapy.

## Breast Cancer Heterogeneity

BC are heterogeneous in nature, both at the histological and molecular levels. Traditional BC treatments initially depend upon the tumor characteristics such as its clinical stage, histopathologic features, and biomarker profiling. Our understanding of its biological characteristics has improved in the last few decades. We can now subtype it with molecular profiling, hormone indicators, growth factor expressions, and many more. The subtyping of BC is still challenging and very volatile. Different stem cell populations and progenitor cells in the mammary gland can cause a paradigm shift in our current understanding of its heterogeneity.

### Histopathologic Heterogeneity

Histological analysis of breast tumors considers its anatomical origin, in most instances, from the junction between the terminal duct and lobule, an area further labeled as “atypical lobules” (Oyama et al., 2000) or hyperplastic enlarged lobular unit (HELU) (Lee et al., 2005). These histologically identifiable lesions are also the earliest precancerous ones reflecting hormone-responsive cancer (Lee et al., 2005). This lesion further exhibits an elevation in estrogen and progesterone receptor (ER-α/PR). Such a histological perspective is essential in chemotherapeutic responsiveness and endocrine therapy study.

**FIGURE 2:**
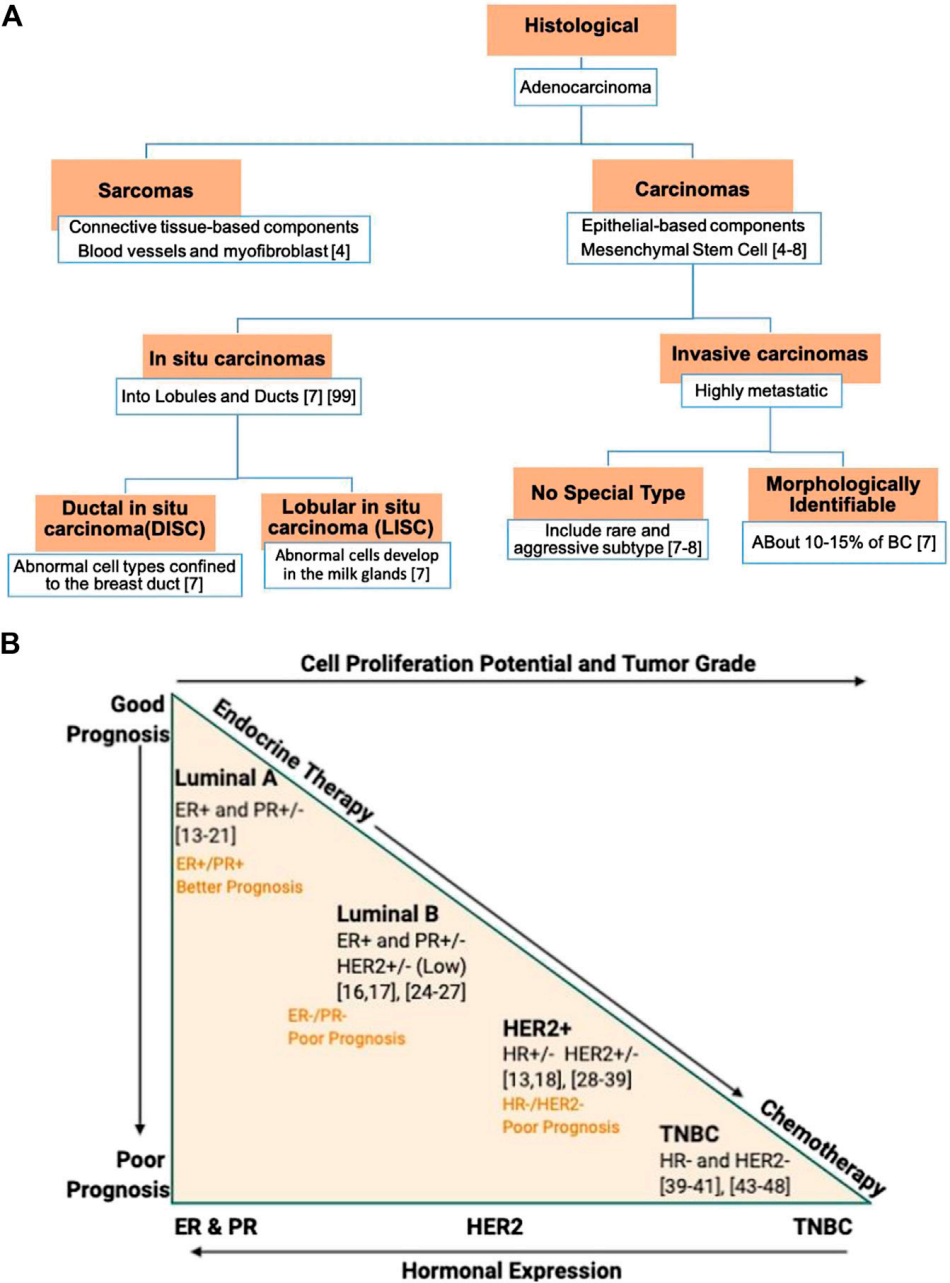
Classification of breast cancer (BC) based on: **(A)** The histopatholgical stratification. **(B)** The molecular stratification, relative grading, therapy requirement, and prognosis. The BC hormone expression reflects an inverse proportion to the tumor grade and cellular proliferation. Luminal A subtype exhibits a better prognosis with a positive response to endocrine therapy. In contrast, TNBC shows no hormonal expression, higher staged and nuclear grade tumor with intense mitotic activity, and poor prognosis. ER, estrogen receptor; HER2, human epidermal growth factor receptor 2; PR, progesterone receptor, and TNBC, triple negative breast cancer.

According to the 2012 WHO classification, BC are primarily categorized into carcinomas and sarcomas (Sinn and Kreipe, 2013) (Figure 2A). If BC’s inception is from the breast’s epithelial cell-based components, including lobules and terminal ducts (responsible for milk), it falls under carcinomas. It further stretches to the underlying mammary stem cells (MSC) that differentiate into epithelial cells (Liu et al., 2005; Shackleton et al., 2006). Unlike carcinomas that usually ascend from milk ducts, sarcoma originates from the connective tissues, such as blood vessels and myofibroblast, which support the ducts and the lobules. It further represents less than 1% of the total BC.

Significant heterogeneity in breast carcinomas further subcategorizes it into *in situ* and invasive carcinomas. The *in situ* carcinomas are more localized to their prevailing lobules and ducts. In contrast, the invasive carcinomas penetrate the neighboring tissues and, if not intercepted, could metastasize to other body tissues and organs. The invasive carcinomas, based on their morphology, are further categorized into morphologically identifiable types and no special type (NST) or “not otherwise specified” (NOS) type. Of them all, the invasive ductal carcinoma (IDC) (Siegel and Jemal, 2015) of NST represents the most frequent type of invasive carcinoma (about 80% of all BC) followed by invasive lobular carcinomas (ILC) of special-type, representing 10–15% of BC. Additionally, ILC growth involves penetration of single cells or cells segregated in sheets, with molecular and genetic aberrations different from IDC. Recently, amongst the rare subtypes of invasive carcinomas, two new entities—tall cell carcinoma with reverse polarity (TCCRP) and mucinous cystadenocarcinoma of NST—have been recognized and listed in 2019 WHO BC’s classification (Hoon Tan et al., 2020). Though they both exhibit tall columnar cell morphology, their core contents are different. Mucinous cystadenocarcinoma of NST contains an abundance of luminal mucin with a cytomorphology of pancreatobiliary and ovarian mucinous cystadenocarcinoma. In comparison, TCCRP represents features like papillary thyroid carcinoma and salivary gland-type tumor. Though they both belong to invasive carcinomas, their malignant potential is low (Hoon Tan et al., 2020).

Additionally, the 3-tier (low, intermediate, and high) grading system further struggles at providing order to the invasive BC-heterogeneity. This grading system analyzes the percentage of tumor in glands and tubular structures (T), degree of nuclear polymorphism or nodes (N), and the mitotic rate (M). However, the stage of a BC is different from its grade. BC staging represents the tumor’s gross appearance, whereas TNM grading allows simplification to BC staging by exhibiting BC’s spread. However, both are heavily incorporated in clinical tools determining the prognosis during BC surgery, such as in Nottingham Prognosis Index (NPI) (Lee and Ellis, 2008).

### Molecular Heterogeneity

Over time, several molecular biomarkers have been reported subtyping BC based on genomic instability (Kronenwett et al., 2006), cytogenetic pathways (Korsching et al., 2002; Hu et al., 2006), gene expression levels (Perou et al., 2000; Kouros-Mehr et al., 2006), and many more. In modern molecular pathology, high-throughput screening on biomarkers provided a highly desired explanation for BC heterogeneity. It delivers biomarkers—estrogen receptors (ER), progesterone receptors (PR), and human epidermal growth factor receptor 2 (HER2) that categorizes BC into five subtypes (Figure 2B): luminal A and B, HER2 enriched, triple-negative or basal-like (BL), and normal-like BC. Stratifying BC will help in expediting the prognosis and treatment selection.

### Estrogen Receptor

ER is the earliest and one of the most prevalent BC biomarkers used (Ellis et al., 2005; Rakha et al., 2010). Many cohorts and cooperative studies with a combined-data set suggest that about 80% of all BC are ER-positive (ER^+^). It is mainly well-differentiated, less aggressive, and great at prognosis than ER-negative (ER^−^) BC (Figure 2B). Based on the stem-cell cancer model, the ER^−^ BC ascends from the most primitive stem cells, where specific mutations limit its differentiation into ER-positive (ER^+^) cells (Prat and Perou, 2009). A broader gene expression profiling (GEP) on approximately 500 genes’ “intrinsic factors” further differentiate ER^+^ BC into luminal-A and -B subtypes with different overall survival (Sorlie et al., 2003). Sorlie and his colleagues observed a high expression of luminal genes and ER^+^ related genes (such as PR) in the luminal-A subtype (Sorlie et al., 2003) than luminal B subtype (Figure 2B). Likewise, the luminal A subtype exhibits a greater prognosis and overall survival than the luminal-B subtype. Consistently, a poor response to endocrine therapies of the luminal-B subtype corroborates with the low ER/PR-expression (Bardou et al., 2003; Creighton et al., 2009; Creighton et al., 2010), high Ki-67 expression (Musgrove and Sutherland, 2009), and an unusual overexpression of HER2 (Ellis et al., 2006) (Figure 2B). As such, Ki-67, the proliferative biomarker, is also suggested as an additional clinical biomarker in differentiating luminal-A from luminal-B subtypes.

### Progesterone Receptor

PR is an ER-regulated gene critical for the lobuloalveolar development of mammary glands (Brisken, 2002). Unlike estrogen and its receptor (ER) that induce ductal outgrowth of mammary glands, progesterone and its receptor (PR) regulate ductal morphogenesis (Atwood et al., 2000). A localized PR cluster stimulates the mammary glands' side-branching by inducing insulin-like growth factor1 (IGF-1) (Ruan et al., 2005). It serves as a negative indicator of tumor aggression in that PR^−^ BC is more aggressive than PR^+^ BC (Cui et al., 2005) (Figure 2B). Thus, both ER and PR are functionally intertwined in mammogenesis, and assessing them together as double receptors will guide the hormonal therapy response.

Taken together, both the receptors have four subclasses under luminal A and B subtypes: ER^+^/PR^+^, ER^+^/PR^−^, ER^−^/PR^−^, and ER^−^/PR^+^. A double-positive subtype—ER^+^/PR^+^—has a better prognosis, and it is more responsive to endocrine therapy (Figure 2B). In a study on subclasses that lack PR expression in the ER^+^ subset, Rakha and colleagues observed a less receptiveness toward endocrine treatment such as tamoxifen (Rakha et al., 2007) compared to the double-positive subtype (ER^+^/PR^+^) (Dowsett et al., 2006). Double negative—ER^−^/PR^−^—exhibits a higher relapse rate with the worst prognosis and overall survival rate. ER^−^/PR^−^ BC acts as an apt candidate for chemotherapy after an unresponsive treatment to endocrine therapy (Bardou et al., 2003). ER^−^/PR^−^ BC can further be stratified based on a third biomarker, HER2 (Sorlie et al., 2003), thus introducing triple receptor classification.

### HER2 Receptor

The BC’s insensitivity to endocrine therapy in a triple receptor classification is rooted in an unusual overexpression of HER2 receptors on mammary glands. It is a transmembrane protein-tyrosine kinase receptor present on normal mammary gland epithelial cells. However, overexpression of about 20%, which establishes genetic instability and excessive proliferation, is regarded as HER2 positive (HER2^+^) BC subtype (Slamon et al., 1989). Also, intimate crosstalk between HER2 and ER/PR signaling pathways corroborates its resistance to endocrine therapies (Schiff et al., 2004; Rakha et al., 2007). This crosstalk excludes ER/PR expression deletion through selective ER modulators (SERM) (Ellis et al., 2001) inhibitors. Similar to luminal A and B subtype, as defined by GEP (Perou et al., 2000), the three receptor-based immune histochemical/compatibility (IHC) evaluation stratifies BC into ER^+^/PR^+^ HER2^+^, ER^−^/PR^−^ HER2^+^, ER^−^/PR^−^ HER2^−^, and ER^+^/PR^+^ HER2^−^, where all HER2^+^ cases shared similar genetic variations (Perou et al., 2000; Cheang et al., 2009) and outcomes (Network, 2012), irrespective of their hormonal subsets (Figure 2B). Preclinical and clinical studies of patients with HER2 BC reports promising results upon merging chemotherapy with anti-HER2 monoclonal antibodies (trastuzumab and pertuzumab) (Piccart-Gebhart et al., 2005) and tyrosine kinase inhibitor (lapatinib and neratinib) based therapies (Cuzick et al., 2011). Furthermore, HER2^+^ BC subtypes, regardless of its ER status, benefit from paclitaxel (a plant alkaloid based chemotherapeutic agent) after adjuvant treatment with an anthracycline-based regimen such as doxorubicin plus cyclophosphamide, specifically in node-positive breast tumors (Hayes et al., 2007; Blum et al., 2017). In conclusion, ER^+^/PR^+^/HER2^+^ BC has the best prognosis and shows an effective treatment response to chemo-hormonal therapy (Cuzick et al., 2011; Lehmann et al., 2011).

### Triple-Negative Breast Cancer

TNBC subtype includes the most aggressive and highly heterogeneous of all BC subtypes. The lack of ER, PR, and HER2 leads to a higher staged nuclear grade cancer with intense mitotic activity and equally poor prognosis (Figure 2B). Due to no hormonal expression, TNBCs are tolerant of endocrine and targeted therapies. Within the last decade, TNBC stratification has been updated frequently. Initially, Lehmann and colleagues, based on GEPs and ontologies from 587 TNBC cases, classified TNBC into six subtypes: BL1, BL2, mesenchymal (M), mesenchymal stem-like (MSL), immunomodulatory (IM), and luminal androgen receptor (LAR) (Lehmann et al., 2011). Whereas, recent findings, based on GEP analysis of most upregulated mRNAs and long non-coding RNAs (lncRNAs) (Liu et al., 2016), merged two subsets IM and MSL into “mesenchymal-like” and Basal1/2 into “BL” to give a most recent classification of four TNBC subtypes (Liu et al., 2016). Though the incursion of LAR assessment into the TNBC subtype requires further investigation, the components involved in the PIK3 pathway is worth considering while developing a targeted therapy (Chia et al., 2015). The IM-TNBC subtype accounts for all the immune-cell associated biomarkers and gene products such as antigen-presenting cells (APCs), chemokines, cytokines signaling components, etc. (Liu et al., 2016). Therefore, in this subtype, targeting immune checkpoints could provide beneficial therapeutic outcome.

Mesenchymal-like TNBC subtype (MES) expresses genes with epithelial-mesenchymal transition (EMT) signature and stem-cell-like properties. It primarily includes cell migration-related signaling pathways such as extracellular matrix-receptor interactions pathways, Wnt pathways, TGFβ signaling, breast stem cells biomarker, ALDH1A1, and other stem cells-oriented genes. It is also called metastatic BC (Lehmann et al., 2011) and is associated with cell differentiation pathways (Lehmann et al., 2011), which could be due to its high motility-related gene expression. Since MES is associated with growth factors, EMT-targeted chemotherapeutic drugs may benefit the patient (Gibson et al., 2005).

BL subtypes are associated at the mammary gland’s basal/myoepithelial level, exhibiting overexpression of cell-proliferative biomarkers such as cell-cycle checkpoints, DNA repair, and replication related genes (Lehmann et al., 2011). Burstein’s reclassification of TNBC subtypes highlights BL subtype exhibiting either downregulation of immune regulating genes—BL-immune-suppressive (BLIS)—and an upregulated immune response—BL-immune activated (BLIA)—TNBC subtype (Burstein et al., 2015). The prognosis index recorded the order in disease-free survival of BL-TNBC subtype—BLIA > M > LAR > BLIS (Burstein et al., 2015). This order could be due to the tumor-infiltrating lymphocytes (TILs) found in the microenvironment of BLIA. The presence of TILs in the BLIA subtype of TNBC could further guide adjuvant chemotherapy treatments. In 2014, the International TILs group proposed facilitating TILs as a stratification factor or one of the significant parameters to assess heterogeneity in BC by hematoxylin and eosin staining evaluation (Salgado et al., 2015).

Traditional BC classifications that include IHC-hormone evaluations, GEP analysis, and examining pathological features have become clinically affordable in routine lab checkups. However, not all transcription synchronizes with the corresponding protein expression. Numerous factors, such as mRNA transcription rate, protein stability, post-translational modifications, and random mutations, affect proteins used as molecular biomarkers. Therefore, for complete knowledge on pathological changes in BC, high-throughput analysis of data extracted from several “omics” studies such as genomic, proteomic, transcriptomic, epigenetics, and Next-Gene Sequencing (NGS)— is needed for analyzing potential biomarkers and pathways involved. However, it still is a long bridge between research findings and its clinical implementations.

### Androgen Receptor

Another potential hormonal receptor—androgen receptor (AR)—is a prevalent sex steroid hormone used in BC subtyping (Labrie et al., 2003). In ER^−^ BC, androgen and its receptor promote cell proliferation and spread (Safarpour et al., 2014) by acting at different components of AR-signaling pathways (Rahim and O’Regan, 2017). The AR is highly expressed in the LAR subtype, with mRNA level nine times or more than any other TNBC subtype (Lehmann et al., 2011), reflecting one-third of TNBCs (Mrklić et al., 2013). IHC analysis also detected a high expression of downstream components of AR-signaling (Mrklić et al., 2013). Therefore, anti-AR therapy is recommended for TNBC patients. In April 2020, the phase II trial showed promising results in its anti-androgen hormone—“bicalutamide”—study in treating metastatic BC patients (updated on ClinicalTrials.gov, Identifier NCT00468715). If the results came through as expected, AR assessment could be integrated into the standard test of TNBC subtypes.

## BC Biomarkers: Established and Promising

**FIGURE 3:**
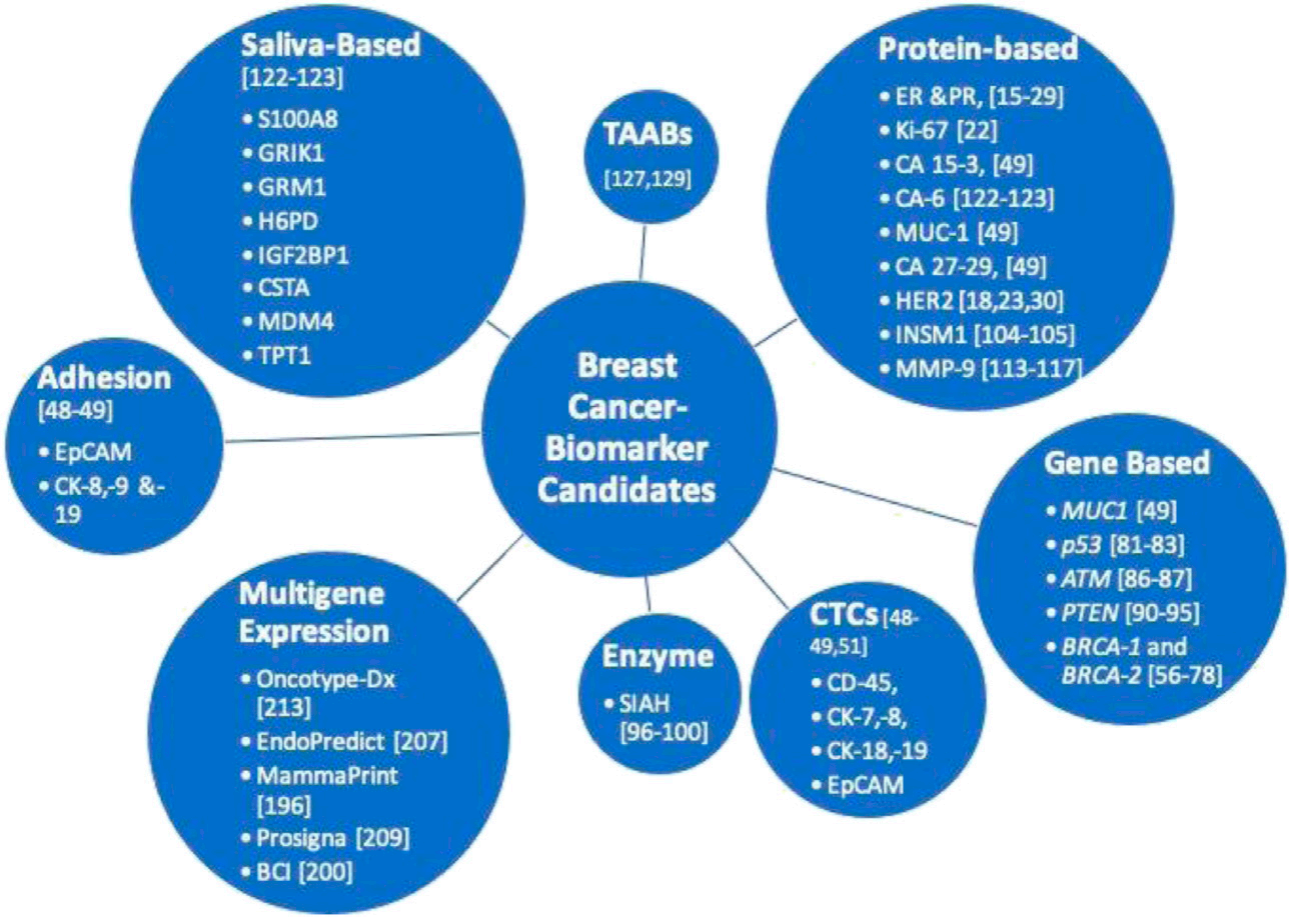
Established and promising breast cancer biomarkers for prognosis and diagnosis.

BC’s systemic management initially considers the expression level of the cell-proliferation gene (Ki67) and hormonal receptors (HR, PR, and HER2) before assessing its subtype clinicopathological and biological parameters. However, various scientific studies also reported some underrepresented single biomarkers (Figure 3). At present, only four such biomarkers—cancer antigens (CA); CA 15-3, CA 27-29, HER2, and circulating tumor cell (CTC) are approved by FDA in assessing BC in body fluids.

The American society of clinical oncology (ASCO) recommends the gene expression analysis of CA 15-3 (mucin1 (MUC1) gene) and CA 27-29 together with bio-imaging for constant monitoring of treatment’s persistence (Van Poznak et al., 2015).

CTCs are approved by the FDA in 2004 to be used in the CellSearch system for measuring and monitoring the metastasis of breast, prostate, and colorectal cancer (Cristofanilli et al., 2007). This CellSearch system analyzes the expression of EpCAM, CD-45, cytokeratin (CK)-8,-18, and CK-19 in body fluids (serum or blood) (Figure 3). In a large cohort of breast carcinoma (575 cases), Shao and colleagues observed that about 90% of all BC exhibit an expression of CK-7. -8, -18, and -19 (Shao et al., 2012). Furthermore, an expression of CK-7 in conjunction with CK-8 showed the utmost sensitivity at detecting BC, especially within high-grade tumors (Shao et al., 2012). Though it promises instant liquid biopsy, the scientific community raises some concerns about the heterogeneity and the low frequency of CTCs, making the detection a bit challenging. However, the addition of more molecular markers such as BSL-2 (Smerage et al., 2013), HER2, EGFR (Zhang L. et al., 2013), CD44, CD47, MET (Baccelli et al., 2013), and many more could assist in rectifying this issue. Nonetheless, this “CellSearch” system is still in its infancy. With a half-life ranging from one to 2 h in BC, CTC fails at guiding subtype-specific therapies (Alix-Panabières and Pantel, 2014).

### BRCA1 and BRCA2

BReast CAncer genes-1/-2 (*BRCA1/2*) are the most common genes implicated in BC risk. Their translated products are phosphoproteins localized in the nucleus (Chen et al., 1996; Bertwistle et al., 1997). BRCA1 protein regulates cellular pathways such as gene-transcription regulation, cell proliferation, DNA repair response, etc., whereas BRCA2 protein regulates DNA repair pathway (Sharan et al., 1997). Early studies on germline mutation in the *BRCA1* gene found that the normal allele or the wild type (WT) copy was deleted in the event of *BRCA*-related cancers (BC or ovarian cancer). The loss of wild type *BRCA1* (or loss of heterozygosity) gene in tumor samples reveals its role as a tumor suppressor gene (Smith et al., 1992). Moreover, Arizti and colleagues observed regulatory parallels between *BRCA1* and tumor suppressor *p53* (Arizti et al., 2000) (Figure 3). Authors further suggested an interesting pathway connecting *p53* and *BRCA1* genes and that their loss under stress conditions could be integral to tumorigenesis (Arizti et al., 2000). In a separate study, loss of function mutation (frameshift or deletion/duplication) in *BRCA1* shown to result in genomic instability with increased susceptibility to malignancy (Deng, 2006). More than sixteen hundred mutations, predominantly frameshift mutations, have been reported so far in the *BRCA1* gene (Godet and Gilkes, 2017). In circumstances where a mutated copy of the *BRCA1/2* gene gets inherited from either parent, the offspring becomes more susceptible to develop BC. However, a single mutated gene doesn’t always result in BC. Only the second mutation or the second defective gene that could affect the wild-type gene triggers BC development. Furthermore, since all cells carry similar genetic imprint, a non-inherited *BRCA* gene mutation is strictly tissue-restricted to the tumor region (breast or ovarian region) (Prevention, 2020; Singh and Yu, 2020). The *BRCA1/2* carriers display a histological characteristic of poorly differentiated high-grade tumors (Musolino et al., 2007). Its metastasis into neighboring vessels indicates a higher risk of contralateral BC (Verhoog et al., 1998; Brekelmans et al., 2007). Mavaddat and his colleagues anticipated the risk to be approximately 83% in *BRCA1* and 62% for *BRCA2* heterozygotes by age 70 (Mavaddat et al., 2013). Though the contralateral prophylactic mastectomy significantly reduced cancer development risk (Van Sprundel et al., 2005), no survival benefit has been observed from the surgery (Brekelmans et al., 2006). Therefore, its early detection and prevention is now the focus of many studies. Upon molecular stratification, approximately 80% of *BRCA1* (Foulkes et al., 2003; Turner and Reis-Filho, 2006) and 3–17% of *BRCA2* related BC belong to the TNBC subtype (Evans et al., 2011) (Couch et al., 2015). Its prevalence also varies among ethnic groups. In Anglian (Anglian Breast Cancer Study Group, 2000) and US non-Hispanic white families (Whittemore et al., 2004), the frequency of pathogenic *BRCA1/2* variants range between 1:400 and 1:500 in the general population, while the highest observed frequency is about 1:40 in the Ashkenazi-Jewish community (King et al., 2003; Dillenburg et al., 2012). With the advancement of technologies such as NGS and multi-gene analysis, knowing the ethnic origin to estimate the chances of mutation(s) in *BRCA1/2* genes is obsolete. However, the knowledge of recurrent mutation in a particular ethnic group could still expedite the diagnosis and treatment among BC patients and related family members (Karami and Mehdipour, 2013), e.g., the three founders *BRCA1/2* congenital mutations account for up to 99% of pathogenic variants amongst the Ashkenazi-Jewish community (Dillenburg et al., 2012). However, the degree of correlation between *BRCA1/2* carriers and BC prognosis is still under investigation (van den Broek et al., 2015).

### Tumor Protein 53

TP53 is a proline-rich tumor suppressor protein first identified on SDS-PAGE an apparent molecular weight of 53 kDa, which later turned out to be 43.7 kDa based on amino acid composition. In human cancers, the *p53 gene* is the most mutated gene that encodes at least 12 TP53 isoforms (p53α, p53β, p53γ, Δ40p53α, Δ40p53β, Δ40p53γ, Δ133p53α, Δ133p53β, Δ133p53γ, Δ160p53α, Δ160p53β, and Δ160p53γ) of varying sizes from 11 exons (Kim and An, 2016). The mutations, most of which are missense mutations, primarily locate in the central DNA binding domain of the TP53 (Marcel et al., 2011), preventing the activity of TP53 by affecting its binding to DNA. Other mutations can yield truncated isoforms which are associated with different cancers. On the other hand, not all of them have their biological significance reported or investigated utterly. In a cohort of 127 BC cases, of three interdependent TP53 isoforms—p53α, p53β, p53γ—, only p53γ isoform displayed a good prognosis similar to its wild type TP53 in BC patients (Bourdon et al., 2011). However, regardless of the mutation, approximately 80% of TNBC cases contain a mutated *p53* gene. And, since TNBC is tolerant to endocrine therapies, mutated TP53 highlights its prospective biomarker role (Figure 3) (Shah et al., 2012). Moreover, the first-in-class monoclonal antibodies developed recently recognizes the most common polymorphic region of TP53 (Hwang et al., 2018), i.e., the DNA binding domain. The antibody displays no cross-reactivity against any other p53 isoform. These mutant-specific monoclonal antibodies hold a great clinical diagnostic potential in targeting minute alterations embedded in various diseased states (Hwang et al., 2018).

### Ataxia Telangiectasia Mutation

*AT* is another tumor suppressor gene, like *p53*, involved in the DNA repair mechanism. Its mutation in women exhibits a greater risk of BC (Thompson et al., 2005). Since it associates with an autosomal recessive disease, patients homozygous for it will be primarily affected. In contrast, the heterozygous patients live an everyday life, but their chances of developing BC are approximately two to four times higher than the general population. An extensive metadata analysis of nineteen heterogeneous cohort studies on relatives of patients suffering from *AT* syndrome suggested that the comparative risk of BC is 6.02% by 50 years of age (95% credible interval: 4.58–7.42%) and 32.83% by 80 years of age (95% credible interval: 24.55–40.43%) (Marabelli et al., 2016) than the general population. Begam and her colleagues also recently concluded their study on aberrant *ATM* promoter methylation as a biomarker to detect BC in patients (Begam et al., 2017) (Figure 3). However, the hurdle in its biomarker role is its relative infrequency of mutation and high prevalence of variants.

### Phosphatase and Tensin Homolog

*PTEN* gene mutation is implicated in a wide variety of sporadic cancers (Milella et al., 2015). It is majorly associated with cellular functions such as genomic stability, cell proliferation, and motility through PI3K dependent/independent pathways. In an invasive BC study on 3,824 patients, an average of 7% exhibited germline mutation in their *PTEN* gene (Gao et al., 2013) (Figure 3). Moreover, although *PTEN’s* mutations aren’t prominent, its loss frequency is approximately 30–40% in BC, accounting for about 25% in HER2^+^ BC (Zhang H. Y. et al., 2013; Veeraraghavan et al., 2017). Furthermore, its insignificant protein expression levels led to investigating/detecting its mRNA levels much more efficiently than its IHC analysis. Likewise, numerous studies have also highlighted the cases where *PTEN*’s loss status fails to correlate with drug treatment response in BC patients. In the tamoxifen plus everolimus (TAMRAD) and the BC Trials of Oral Everolimus-2 (BOLERO-2) studies, *PTEN*’s status fails to correlate with the response to everolimus treatment in BC patients (Treilleux et al., 2015). Moreover, studies on HER2^+^ BC further reported an unsuccessful association between *PTEN* loss and anti-HER therapy response (trastuzumab and lapatinib) (Fujita et al., 2006; Nuciforo et al., 2015). Therefore, clinicians should also combine other pathological parameters of BC besides analyzing *PTEN* gene expression.

### Seven in Absentia Homolog

A growth factor activated RAS pathway is responsible for uncontrolled cell growth, proliferation, and dissemination in various human cancers (Schmidt et al., 2007). In BC, the RAS pathway is an understudied pathway due to an insignificant detection of RAS mutations in mammary tumors (only in about 5% of the BC patients) (Arteaga et al., 2012). Further, it has been observed that the most downstream and an essential component of the EGFR/HER2/RAS pathway is an evolutionarily conserved E3 ligase— SIAH—that acts as a “gatekeeper” to tumorigenesis (Medhioub et al., 2000; Schmidt et al., 2007). Behling and colleagues found that its expression was proportional to the progression of ductal carcinoma *in situ* (DCIS) to invasive carcinoma (Behling et al., 2011). Though it is the most downstream component of the pathway, inhibiting SIAH expression or its enzymatic activity inhibits the RAS-mediated tumor growth and metastasis in nude mice (Schmidt et al., 2007). The inhibitory effect of reduced SIAH expression may affect the upstream of the pathway, as a feedback loop mechanism. Its enzymatic activity may be nurturing/fostering the tumor cells by modulating its microenvironment. Clinically, it can be recognized in combination with EGFR or alone as a surrogate biomarker guiding chemotherapy treatment by analyzing the depth and recurrence of chemo-resistant tumor clones (van Reesema et al., 2016). Its on and off expression reflects the aggression and repression in post-neoadjuvant chemotherapy patients, a prognostics that outperform the HR/HER/Ki67 as a new biomarker (van Reesema et al., 2016) (Figure 3). Furthermore, based on its expression and enzymatic alterations, expectations are to develop a targeted therapy against SIAH, alone or in combination with EGFR, for chemo-resistant, relapsed, late-stage, and metastatic BC.

SIAH is an evolutionarily conserved gene, and its mutations rarely account for any specific disease (Medhioub et al., 2000; Schmidt et al., 2007; Zhao et al., 2016). Some research groups have observed *SIAH1* gene mutations in certain carcinomas such as hepatocellular carcinoma, prostate cancers, breast cancers, etc., whereas the results were hard to interpret because of the interference of other tumor suppressor genes located on the same chromosome (Medhioub et al., 2000; Zhao et al., 2016).

### Insulinoma-Associated Protein 1

In 2003, the WHO incorporated a classification of BC with neuroendocrine (NE) differentiation features (Lakhani et al., 2012), which was first reported in 1963 for its correlation with BC. The classification was further revised to carcinomas of neuroendocrine features in the 2012 WHO classification of BC (Bussolati and Badve, 2012; Lakhani et al., 2012). Its subgroup, invasive breast carcinoma (IBC) with neuroendocrine differentiation (IBC-NE), is a rare subtype predominant in postmenopausal women. In a large IBC cohort, Razvi and his colleagues evaluated a biomarker—INSM1—for NE differentiation using IHC (Razvi et al., 2020). According to the authors, in about 7% of the cases, the INSM1-IHC expression profile was found comparable or more sensitive than predefined NE biomarkers: chromogranin A and CD56, but less sensitive than synaptophysin (Razvi et al., 2020). INSM1 was initially reported, by Goto and colleagues, to be in the fetal pancreas and nervous system as a zinc finger transcription factor (Goto et al., 1992). Most recently, it has been observed in high grade and aggressive breast carcinomas, particularly among luminal-B subtypes (Wachter et al., 2014; Razvi et al., 2020). The authors further suggested INSM1 expression as a favorable prognostic biomarker (Figure 3), which could be useful in stratifying NE-tumors (NET) with different prognosis (Razvi et al., 2020).

### Matrix Metalloproteinase-9

Matrix metalloproteinases (MMP) are a family of endopeptidases acting on a broad range of proteins such as gelatin, collagen, and elastin (Kessenbrock et al., 2010). MMP-9, also known as gelatinase B, is an extracellular protease that remodels the tumor environment by degrading the endothelial basement membrane (Kessenbrock et al., 2010). The disrupted membrane enables carcinoma invasion and triggers angiogenic switch, a necessary step in tumor progression (Gialeli et al., 2011; Mehner et al., 2014). MMP-9 also activates soluble factors such as cytokines, which induce EMT and invade microenvironment of distant organs, promoting metastasis (Gialeli et al., 2011; Mehner et al., 2014). Its expression is regulated by various pathways, such as MAPK, ERK, EGFR/PI3K (Dziembowska and Wlodarczyk, 2012; Shi et al., 2015), implicated widely in BC. MMP-9 is considered as a potential biomarker in various cancers (Tian et al., 2008; Li et al., 2012; Li L.-N. et al., 2013; Blanco-Prieto et al., 2017; Chen et al., 2018; Zhou et al., 2018) (Figure 3). In BC, MMP-9 expression varies with its molecular (intrinsic) subtypes (Yousef et al., 2014). Yousef and colleagues observed a signature expression of MMP-9 in HER2^−^ and TNBC subtype with node-positive breast carcinoma (Yousef et al., 2014). Multivariate serum analysis for MMP-9, together with the extracellular domain (ECD) of HER2 (HER2-ECD) and neuron-specific enolase (NSE) (a non-specific NE biomarker), was able to discriminate between BC patients for brain metastasis (Darlix et al., 2016). Furthermore, a non-invasive multivariate exploration could stratify BC patients based on serum MMP-9 expression in conjunction with Rho expression in circulating leukocytes (Golubnitschaja et al., 2017) for BC risk assessment.

MMP-9 polymorphisms have been associated with many diseases such as pancreatic ductal adenocarcinoma (Tian et al., 2008), ovarian and cervical cancer (Li et al., 2012; Li L.-N. et al., 2013), lung cancer (Blanco-Prieto et al., 2017), bone tumor (Varn et al., 2017; Chen et al., 2018), atherosclerosis. However, its relationship with BC’s occurrence is still unclear (Felizi et al., 2018).

### Salivary Biomarkers

Investigating the saliva’s protein profile for discriminatory cancer biomarkers has been shelved for decades due to technological limitations. Recently, salivary metabolite profiling has received much attention as a noninvasive biomarker for early BC detection. Zhang and colleagues (Streckfus et al., 2006) initiated a *de novo* biomarker discovery approach in saliva (Zhang et al., 2010). The study established a total of nine biomarkers, eight mRNAs (S100A8, GRIK1, GRM1, H6PD, IGF2BP1, CSTA, MDM4, and TPT1) and one CA protein-6 (CA-6), with a clinical diagnostic accuracy of 92% (Zhang et al., 2010) (Figure 3). The protein, CA-6, was also found to be a marker in an earlier study on saliva samples of DCIS patients (Streckfus et al., 2006). Subsequent studies found an altered metabolism for approximately 28 different metabolites (Sugimoto et al., 2010), such as valine, proline (Cheng et al., 2015), taurine, lysine, and sialic acid (Ozturk et al., 2011), statistically discriminating BC from healthy controls. Moreover, Laidi and colleagues suggested that salivary autoantibodies could play a role in BC screening (Laidi et al., 2016). In conclusion, saliva-based biomolecules have shown growing importance in salivary biomarker discovery for future research. However, ASCO has not yet endorsed the use of salivary biomarkers as diagnostic tools for BC.

### Tumor-Associated Autoantibodies

TAABs are antibodies produced by a patient’s immune cells against tumor-associated antigens (TAAs) that are comparatively overexpressed in cancer cells (Laidi et al., 2016). The body’s immune system recognizes TAAs as foreign entities and triggers an acquired immune response to produce TAABs. These antibodies are an amplified “tumor signal” that may fulfill the biomarker features of cancer, i.e., specificity and sensitivity (Figure 3). With the advancement in technologies, such as serological proteome assay (SERPA), serological analysis of tumor antigens by recombinant DNA (cDNA) expression cloning (SEREX), multiple affinity protein profiling (MAPPing), and many more, novel immune biomarkers of BC have been discovered and utilized in the early detection of antigens such as TP53, HSP60, Mn-SOD, cyc-B1, c-myc, and so forth (Hamrita et al., 2008). However, these TAAs are aberrantly expressed or post-translationally modified or irregularly regulated in tumors. It is, therefore, evident that a single TAA-targeted TAAB isn’t sufficient for BC detection. A set of multiple TAA-targeted TAABs followed by validation through traditional techniques such as enzyme-linked immunosorbent assay (ELISA), antigen array, and many more could discriminate cancer cells against healthy control. Kim and colleagues successfully devised an autoantibody-based bead array panel of 35 TAAs in a multiplexing assay with an accuracy of approximately 91% (Kim et al., 2009). Noninvasive and simplified detection of TAABs paved the way for its future research as diagnostic biomarkers. Although these TAABs show a strong titer, their heterogeneous nature—due to TAAs’ post-translational modification (PTM)—and our inadequate understanding of humoral response, limit their clinical applications.

## Breast Cancer Diagnostics

### Mammography

Traditionally, mammography has been used as a gold standard in the screening of BC (Society, 2019). It is a low dosed X-ray exam for each breast, examining breast lumps, skin changes, and nipple discharge or thickening. The digital images, known as a mammogram, are taken both horizontally and vertically to cover a breast to its entirety. These mammograms interpret for mass/lesions, calcifications, and architectural distortions in the breast tissue (Barazi and Gunduru, 2019). Mammography detects such mass/lesions relatively at a stage when the mass has already progressed into a tumor. The interpretations are reported in a Breast Imaging Reporting and Data System (BI-RADS) to communicate with the physician for further assessments (Reporting, 2003). Despite being the gold standard in breast screening, it has significant limitations. The rate of false-positive is significantly high (Hubbard et al., 2011). An increase in fibro-glandular breast tissue density increases the chance of masking or mimicking the underlying BC in a mammogram because both dense tissue and cancer appear white (Boyd et al., 2007). Also, women may fail to inform about their breast implants, leading to misidentification (Society, 2019). Moreover, though a low dose, radiation generated health concerns among patients (Hauge et al., 2014). Out of these limitations, clinical trials are in session evaluating if mammography can be substituted by other advanced techniques such as MRI (Moy et al., 2009) or digital breast tomosynthesis (DBT) (Society, 2019). DCIS or lobular carcinoma *in situ* (LCIS) and IDC, mammography is the best, superior to MRI and ultrasound. Yet, for detecting ILC, a biopsy is more suitable than mammography (Society, 2019).

### Molecular Imaging

Magnetic Resonance Imaging (MRI) is a standard technique that uses a magnetic field, in contrast to X-rays in mammography, to analyze the lumps in the breast that later need to be biopsied (Society, 2019). Several modifications have been incorporated in MRI over decades. For example, a contrast reagent, such as gadolinium diethylenetriamine penta-acetic acid (GD-DTPA), is injected into the bloodstream, followed by MRI detection to enhance the images in pinpointing the suspected area for further analysis. Similarly, radioactive chemicals (in Breast Specific Gamma Imaging (BSGI) or Scintimammography) or radioactive particle conjugated with a sugar moiety (in Positron Emission Mammography (PEM)) can be injected intravenously to study the spread of BC and to follow up on patients. With technological advancement in MRI based techniques such as dynamic contrast-enhanced MRI (DCE-MRI) (Chang et al., 2016; Rahbar and Partridge, 2016), chemical exchange saturation transfer MRI (CEST-MRI) (Cai et al., 2014), etc., evaluating metabolic heterogeneity within tumors becomes feasible. However, the requirement of sophisticated equipment constraints its availability to all hospitals/clinics. The exposure to radioactive materials and expenses involved further limit a patient’s willingness to a yearly checkup. As a result, they are recommended only to high-risk women patients (Society, 2019).

### Ultrasound

As a complementary procedure, ultrasound is a follow-up examination to confirm a positive mammogram (Society, 2019). Several reports have supported the use of automated breast (AB) ultrasonography (ABUS) over mammography for dense breast tissue exams (Kolb et al., 2002; Society, 2019). Contrast-enhanced ultrasonography (CEUS) and microvascular imaging successfully discriminate malignant and benign tumors by analyzing blood flow dynamics in local tissues (Li Y.-J. et al., 2013). CEUS further guides or predicts the effects of neoadjuvant chemotherapy on BC patients (Amioka et al., 2016). As a supplement to mammography, ABUS identifies an additional 1.9 cancer cases per 1,000 women screened (Brem et al., 2015). However, it requires a quality interpretation by an experienced radiologist to minimize false positives.

### Digital Imaging/Spectroscopy

Recently, three new noninvasive technology have emerged promoting BC screening and diagnosis—digital infra-red thermal imaging (DITI), digital image-based elasto-tomography (DIET), and electrical impedance scanning/spectroscopy (EIS).

DITI measures a localized skin temperature difference to reflect the physiological changes such as vasodilation, neovascularization, inflammation, lymph dysfunction/congestion (Anbar, 1998), and other suspicious activities around the breast borders (sternum or axilla). The changes recorded by the infra-red camera are graphed into a heat map of the breast, also called a thermogram. However, due to its low accuracy, its clinical application is limited. With the recent development of a high-resolution infrared camera, a new interest has been generated in its usage as a BC detection tool.

DIET utilizes the vibrational energy in graphing the location of the tumor (Lee et al., 2014). Sinusoidal waves of low-frequency vibrations (5–100 Hz), as a result of surface motion, are induced in the breast, and the oscillations are recorded by digital cameras tracking fiducial markers (Brown et al., 2007; Peters et al., 2009). Areas with a different surface vibrational response compared to control tissues are considered potentially the tumor infected areas. Feeding these responses to an algorithm allows an evaluation of the phase delay in surface-vibrations, thereby detecting the tumor’s angular location, depth, and size (Peters et al., 2008). Clinically, it can detect small tumors that may have gone undetected by an experienced clinician, who depends on manual palpation as the initial diagnosis. Moreover, it can reduce false positives as a screening method, thereby preventing unnecessary radiation and discomfort. However, it cannot provide information on the subtype of BC involved (Ganau et al., 2015).

EIS measures the electro resistance (bio-impedance) (Jossinet, 1996) with the principle that tissues have different electrical properties under different metabolic conditions (Morimoto et al., 1990). The multi-frequency EIS measures the tissue’s overall bio-impedance/resistance by varying frequencies (50 samples), ranging from 300 Hz to 10 MHz (Jossinet, 1996). Since a disease (like cancer) creates finite metabolic changes in tissues, EIS can discriminate diseased tissues from normal tissues by analyzing the amount of impedance contributed by different cellular components, including the extracellular environment (Alzurq et al., 2016). For example, at low frequency (<1000 Hz), an electric current will pass through the extracellular fluid only, but at higher frequencies (10 kHz–10 MHz), it can conduct through both intracellular and extracellular spaces (Morimoto et al., 1990; Jossinet, 1996; Chauveau et al., 1999). Clinically, the EIS spectrum of higher frequencies is found more relevant at detecting malignancies (Ollmar and Grant, 2016). Using EIS, Haeri and colleagues successfully separated BC patients from healthy patients by accurately measuring the cell shape, structure, depth, intracellular and extracellular environment, and lipid membrane compositions (Haeri et al., 2016).

### Biosensors

**FIGURE 4:**
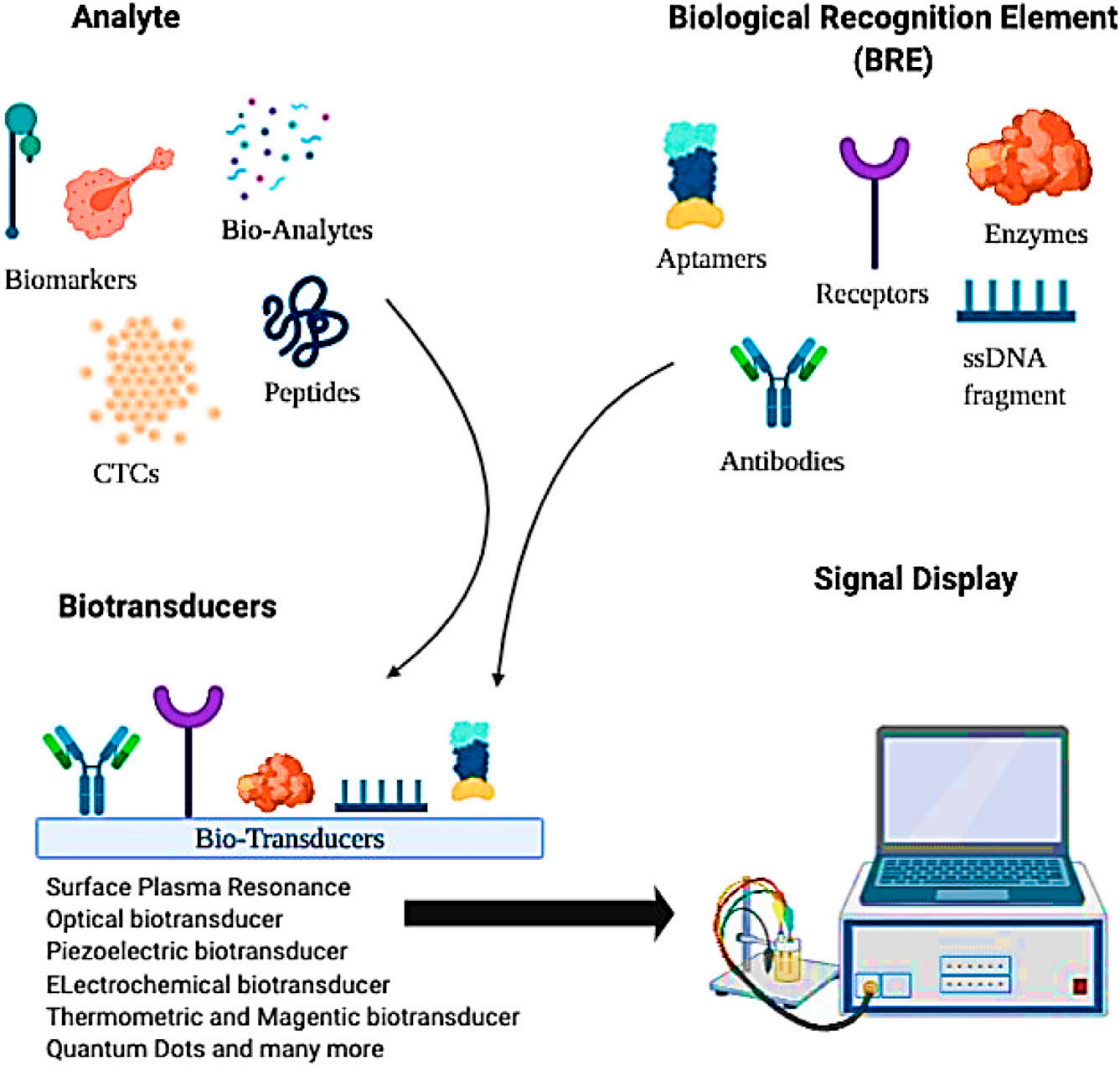
A schematic representation of a simple biosensor in detecting specific biomolecules such as cancer-specific biomarkers, shed-off extracellular domains, circulating tumor cells, etc., extracted from different body fluids.

Biosensors’ inception can be traced back to Leyland’s lab in 1956, where the Clark electrodes were invented to detect blood-oxygen (Clark, 1956). Since then, remarkable modifications have been documented in biosensors to sense any abnormality that could result in disease. Biosensors consist of three key components: a receptor, a biomarker, and a transducer (Figure 4). Besides cell surface biomarkers, their shed off ECDs also act as potential biomarkers (Marques et al., 2014). Multiple bioreceptor—biochemical recognition elements (BRE)—are employed to detect such biomolecules in a sample. Biotransducer converts positive interactions between biomarker and bioreceptors into measurable signals (Marques et al., 2014). An adequately structured biosensor is portable in nature with user-friendly features. Its non-invasive and inexpensive point-of-care analysis provides a quick response without compromising its specificity and selectivity (Nikhil et al., 2016).

In a biosensor, BRE is the most versatile component. It could be any entity with specificity toward a biomarker such as antibodies, aptamers, enzymes, CTCs, and many more (Figure 4). In this regard, antibodies are the most common BREs used in biosensors. Contributing to real nano-sense at detecting BC, antibodies have created a commercial niche in BC diagnosis. With recombinant antibodies—a third-generation antibodies—that contains modified antigen-binding domains, the sensitivity limitations that were previously associated with traditional—poly- or monoclonal—antibodies, can now be overcome (Haurum, 2006). It could detect BC biomarkers directly (Sonuç and Sezgintürk, 2014; Eletxigerra et al., 2015) or indirectly by using enzymatic probes (for example, HRP) (Yang et al., 2011), fluorescence probes (Chang et al., 2011) or cross-linkers (Wang et al., 2014; Arkan et al., 2015). However, the thermal instability of antibodies and the tests’ reproducibility are still a challenge and need further research.

Researchers have designed a string of specific nucleotide/peptide sequences, which function on the same principle as antibodies but one-third of the size, as tight target binders known as aptamers. Peptide aptamers and nucleic acid aptamers (NAAs) can bind specifically with high affinity to biomolecules, circulating in body fluids, such as glycoproteins, microRNA (Won et al., 2013), and most recently to whole cells (Rong et al., 2016). Interestingly, nucleotide aptamers are selected from a large DNA/RNA library through a combinatorial process—systemic evolution of ligands by exponential enrichment (SELEX)—used in molecular biology for producing ligand-specific oligonucleotides (Tuerk and Gold, 1990). Aptamers bind because they “fit” their targets. With remarkable folding properties, aptamers make secondary and tertiary structures of ssDNA or RNA interacting with the BC biomarkers (Chambers et al., 2008). Its binding to the target is determined by how the bases are stacked, the intercalations, and the target’s hydrophobic interactions. These physical parameters can be chemically refined to bind a variety of targets with specificity. Unlike antibodies, aptamers are easily chemically modifiable by conjugation chemistry to provide greater thermal stability and reduced toxicity. Several researchers have designed aptamer for HER2 (Chun et al., 2013; Qureshi et al., 2015), EpCAM (Song et al., 2013), MUC1 (He et al., 2012; Hu et al., 2014), VEGF (Zhao et al., 2011) and nucleolin (Feng et al., 2011) for early BC detection.

Besides aptamers and antibodies, cDNA based hybridization biosensor has been investigated in various BC detection studies (Figure 4). In one study, the detection of microRNAs (Zhang et al., 2016) and the *BRCA1* gene (Cui et al., 2017) have been permitted using a modified cDNA hybridization technique. The hybridized complex formed between cDNA and the target was further amplified in real-time by a DNA polymerase enzyme, right at the electrode. The authors additionally observed an exponential increase in the signal, which shortens the analysis time while keeping the sensitivity and selectivity intact (Benvidi et al., 2015).

Similarly, compatibility between BRE and biotransducer is of utmost importance for a useful biosensor. Several modifications have been made in biotransducers to quantify the acquired signal in proportion to the analyte concentration (Nikhil et al., 2016). Modifications such as, optical transducers (Dey and Goswami, 2011), electrochemical (Grieshaber et al., 2008), piezoelectric biotransducers (Pohanka, 2018), thermometric and magnetic-based transducers etc. have been employed by various researchers for targeting *BRCA1* (Culha et al., 2004; Benvidi et al., 2015), HER-2 (Chang et al., 2011; Marques et al., 2014), MUC1 (Chang et al., 2011; Hu et al., 2014), miR-21 (Torrente-Rodríguez et al., 2015; Li D. et al., 2016), EpCAM (Arya et al., 2013), etc., in early BC detection. Also, nanoparticle enhancers were employed alongside with biotransducers to generate an amplified readout signal. Surface plasmon resonance (SPR) biotransducers that provide real-time sensing by analyzing the changes in refractive index upon interaction with labeled biomolecules has recently been used in conjunction with nanoparticles, resulting in the enhancement of the sensitivity of detecting biomarkers such as CA15-3 (Liang et al., 2012), HR (Neo et al., 2009), and MUC1 (Li Y. et al., 2016). In the past few years, research in quantum dots (QDs)/nanocrystals has gained momentum in early BC detection. QDs as nano-labels, structurally, provide a better platform for antibody/protein/aptamer conjugation that can target biomolecules such as tumor-associated exosomes (Boriachek et al., 2017), CTCs (Wu et al., 2018), etc. In a study, Cheng and colleagues designed a three-component DNA construct containing—MUC1 multi-peptide aptamer stem, QDs-reporter, and a quencher—that detects MUC1 peptide at a nano-molar level (Cheng et al., 2009). Freitas and colleagues successfully screened HER2¯ECD biomarkers in human serum by combining QDs to electrochemical immunosensing (Freitas et al., 2020). The entire screening was completed within 2 h, with hands-on-time of less than 30 min (Freitas et al., 2020). Compared to enzyme-based label systems, the QD label electrochemical sensing eliminates the substrate requirement, surpasses enzymes’ thermal instability, and provides quick analysis.

In conclusion, by offering a quick, simple, and inexpensive diagnostic tool, especially in BC, the biosensor market is growing exponentially and is expected to surpass approximately US$40 billion by 2022 (Pfeifer, 2018). Despite all that, further research is needed to increase the detection sensitivity in almost all biosensors.

## Advanced BC Detection Systems

### Multiple Gene Prognostic Assays

In the era of big data, analyzing the large number of sequences (DNA, RNA, and protein array) from various studies can help devise a systemic strategy combating BC. Several genetic aberrations in women have a predisposition to BC. Incorporating genes that have been implicated in BC as a potential biomarker can provide an advantage in early BC detection and treatment (Walsh et al., 2011). With multigene signature analysis alongside other molecular tools, clinicians can now devise appropriate therapies and predict their outcome while minimizing detrimental effects. Currently, there are seven prognostic multigene signature analyzing tests for BC. However, not all are FDA approved. Some are recommended by agencies, such as the ASCO, American-National Comprehensive Cancer Network (NCCN), and the European Society of Medical Oncology (ESMO) in their guidelines.

### Breast Cancer Index Test

BCI test is a multigene assay developed by Biotheranostics Inc. It records: 1) a set of five proliferative gene expressions (*BUB1B, CENPA, NEK2, RACGAP1,* and *RRM2*), termed as molecular grade index (MGI), and 2) a gene ratio of *HOXB13: IL17BR* (H: I), in predicting the BC outcome (Sgroi et al., 2013) (Table 1). The study compared BCI and IHC4 for their predictive ability in early and late recurrence in postmenopausal-ER^+^ patients with node-negative BC who participated in the clinical trial for arimidex, tamoxifen, alone or in combination (Sgroi et al., 2013). The linear BCI model showed significant prognostic value for risk of both early and late distant recurrence. Consistent results were reported in the Stockholm TAM cohort (Zhang et al., 2013), where BCI was able to identify two risk populations in both early and late distant recurrence and aiding in residual risk management after 5 years. In ASCO 2020 meeting, Biotheranostics’s Trans-aTTOM study delivered more evidence on BCI as a powerful prognostic tool informing the risk-benefit for extending adjuvant therapy (Bartlett et al., 2020). However, the BCI scores can only be used as an adjunct tool to the operating physician’s practice because it relies on the correlation with the patient’s clinicopathological study. In conclusion, though its implications are worth considering in clinical research, it has still not been approved by the FDA.

**TABLE 1:** List of genes/proteins common in the different multigene test analysis. All abbreviations are widely known and are procured following the HGNC and IPNC guidelines. Color coding reflects common genes/proteins included in multiple testing panels. 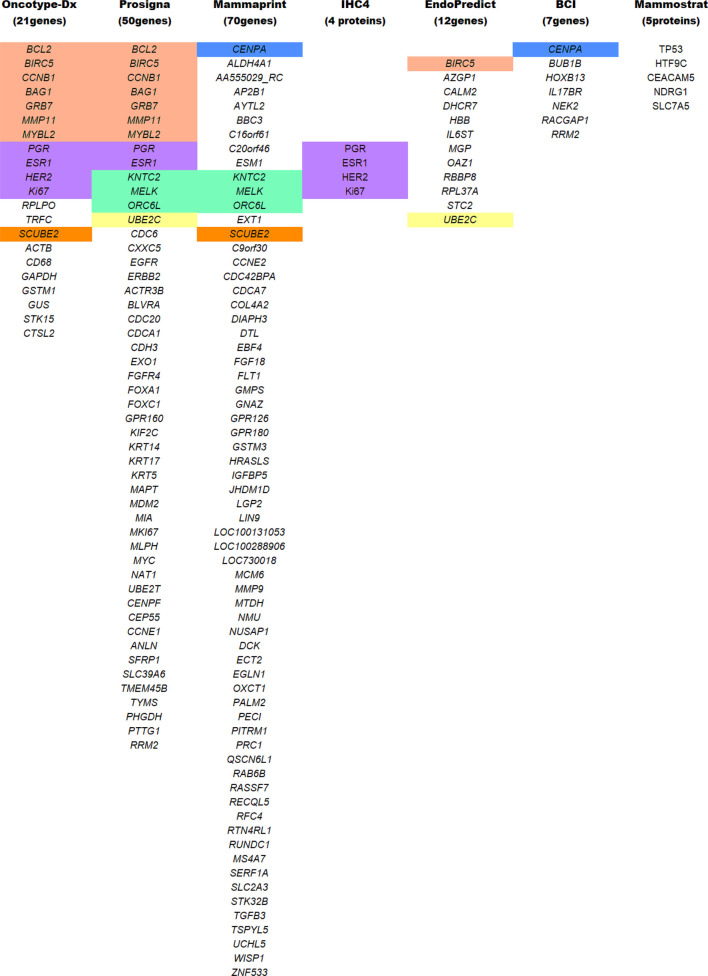

### MammaPrint Test

The MammaPrint test developed by Agendia is a genomic test used for early-stage BC diagnosis. NCCN, ESMO, and ASCO have recommended molecular signature analysis of 70 genes for primary BC prognosis (Cardoso et al., 2016) (Table 1). Its FDA’s approval as a prognostic test stratifies early-staged hormonal BC into low vs. high-risk for relapse (Van De Vijver et al., 2002). The ASCO guidelines of 2017 incorporate MammaPrint test scores to guide on therapy required for high-risk HR^+^/HER2^−^ with node-negative breast carcinoma (Krop et al., 2017). The guidelines further state that “*MammaPrint assay may assist in decisions on withholding the therapy in patients with one to three positive nodes and high clinical risk, provided that the patient should be informed that a benefit from chemotherapy cannot be excluded*” (Krop et al., 2017). Extensive validation studies were performed before the MammaPrint tool became the standard of care. The Microarray In Node negative disease may Avoid Chemotherapy (MINDACT) study on 6,693 women with early-stage BC observed that the 70-genes analysis was able to detect even small aggressive tumors and was able to spare chemotherapy in patients with high clinical risk and low genomic risk of recurrence (ROR) (Cardoso et al., 2016). Therefore, the circumvention of chemotherapy in postmenopausal women with a low-risk of recurrence (as per the MammaPrint test) showcases its clinical utility as a prognostic genomic test (Cardoso et al., 2020). However, in TNBC and HER2^+^ BC, MammaPrint tests are not recommended until additional studies and results met the ASCO requirements.

### Mammostrat Test

In contrast to multigene tests, Mammostrat is a five-protein based IHC assay (Table 1). It analyzes proteins (TP53, NDRG1, CEACAM5, HTF9C, and SLC7A5) that have been implicated in BC recurrence (Ring et al., 2006). The test score stratifies early-staged luminal BC with node-negative or positive carcinoma into low-risk to high-risk patients (Bartlett et al., 2010). Mammostrat test evaluates early-stage BC patients on tamoxifen therapy, analyzing and informing them of adjuvant therapy’s benefits. Like BCI, it is an adjuvant factor for BC evaluation. As per ASCO guidelines, patients with HR^+^/HER2^−^ with node-positive or negative breast carcinoma are moderately not recommended for the Mammostrat test. But, for TNBC or HER2^+^ patients, the Mammostrat test is strictly not recommended.

### IHC4 Test

IHC4 is a hormonal receptor protein-based assay focuses on four primary proteins, ER, PR, HER2, and Ki67 expressions, which were previously used as biomarkers to define surrogate molecular subtypes. Unlike BCI, IHC4 evaluates protein expressions, none of which is translated by BCI associated genes. As an independent tool, IHC4 combines the information from four biomarker proteins (Cuzick et al., 2011) to predict patients’ prognosis (Table 1). A modified version of IHC4 (mIHC4) was recently suggested for ER^+^/HER2^−^ metastatic BC patients (Jin et al., 2020), guiding on chemo or endocrine therapy. However, IHC4 is not recommended for TNBC and HER2 carcinoma (Harris et al., 2016), and the ASCO guidelines have not endorsed the use of IHC4 due to its unsatisfactory reproducibility (Cuzick et al., 2011).

### EndoPredict Test

EP is a twelve multigene signature assay developed by Myriad Genetics (Table 1). It examines biopsied tumor tissue for eight cancer-related genes, three RNA-reference gene, and one DNA-reference gene (Filipits et al., 2011). The test score (EPclin Risk Score) that reflects tumor size and nodal status stratifies BC as low or high risk for distant metastasis (Filipits et al., 2012). In the GEICAM trail, EPclin Risk Score was used as an independent prognostic parameter for ER^+^/HER2^−^ BC with node-negative patients treated with chemotherapy followed by hormonal therapy (Martin et al., 2014). According to the ASCO guidelines, EPclin Risk Score may also be employed in the decision-making process as an adjuvant factor in ER^+^/PR^+^ HER2^−^ node-negative BC patients. However, for HER2^+^ and TNBC with node-positive breast carcinoma, ASCO doesn’t recommend EPclin Risk Score due to insufficient evidence for its usefulness (Harris et al., 2016).

### Prosigna/Prediction Analysis of Microarray50 Test

Prosigna, formerly known as Prediction Analysis of Microarray50 (PAM50), is a RNA-based gene molecular signature assay developed by NanoString Technologies (Martin et al., 2014). This assay helps profile a patient’s tumor and understand its behavior. It includes analysis models to evaluates relapse/recurrence based on BC’s intrinsic hormonal subtyping, developed by Parker et al. (Parker et al., 2009). In Prosigna, the RNA extracted from the biopsied tumor sample is analyzed to provide information on the subtype involved and predict the tumor’s recurrence. The analysis is based on the Prediction Analysis of Microarray (PAM) signature assay (Martin et al., 2014) (Table 1), evaluating the activity of 58 genes by integrating NanoString’s nCounter technology to simplify the workflow without compromising its efficiency (Nielsen et al., 2014). The fluorescent probes used in nCounter technology bypasses mRNA amplification, thus saving time and material. The results are digitalized and later fed into an algorithm that translates tumor biology into actionable clinical results (Tibshirani et al., 2002). The algorithm includes hormonal expression and clinicopathological assessment of tumor based on its size and proliferative potential. The results scaled on 0–100, as the risk of relapse/recurrence (ROR) score, classifying node-negative cancers into low (0–40), intermediate (41–60), or high (61–100) risk, and node-positive cancers into low (0–40) or high (41–100) risk. According to the ASCO guidelines, a physician may use this signature assay alongside the clinicopathological parameters for high-risk HR^+^/HER2^−^ node-negative BC patients to select an adjuvant therapy (Harris et al., 2016). However, it doesn’t recommend using Prosigna score for the low-risk group, HER2^+^and TNBC with node-positive breast carcinoma (Harris et al., 2016).

### Oncotype Dx Test

Oncotype Dx is one of the most validated multigene signature assay, developed by Paik and colleagues (Paik et al., 2004) at Genomic Health, Inc. The ASCO guidelines incorporate it for the early-stage ER^+^/HER2^−−^ node-negative BC patients with a high risk of recurrence Tian et al. (2008). It analyzes the RNA expression of 21 genes implicated in cancer proliferation and treatment response Tian et al. (2008) (Table 1). In conjunction with the patient’s age, the algorithm calculates a score between 0 and 100 as an Oncotype Dx Recurrence Score (RS), stratifying early-stage BC into a group of low, intermediate, and high risk of recurrence. In the low risk (RS < 18) group, chemotherapy benefits outweigh the risk of side effects, wherein high risk (RS > 31) group, chemotherapy benefits should surpass the rise of side effects. In this manner, the intermediate (RS 18-31) groups are the most volatile ones, with uncertainty whether chemotherapy outweighs the side effects or not. With recent technological advancement and research in GEP, an optimization was made in the RS cutoff (Sparano et al., 2015). In the Tailor-Dx phase-3 trial study, Sparano and colleagues confirmed its usefulness in guiding adjuvant systemic therapy (Sparano et al., 2015) in women older than 50 years. According to the authors, chemotherapy can be avoided in 1) women older than 50 years with RS 11-15, 2) women younger than 50-year-old with RS 11-16, and 3) women with RS 0-10. Chemotherapy followed by hormonal therapy were assigned for women with RS > 25. ASCO guidelines advise against the application of chemotherapy for the early-staged HR^+^/HER2^−^ node-negative BC patients with low risk of recurrence. The guidelines provide no direct assessment in intermediate RS patients; instead, they refer to TAILORx’s recommendation alongside traditional prognostic factors (Andre et al., 2019). The guidelines further indicate that the Oncotype Dx test should not be used in HER2^+^ and TNBC (Harris et al., 2016; Krop et al., 2017).

**FIGURE 5:**
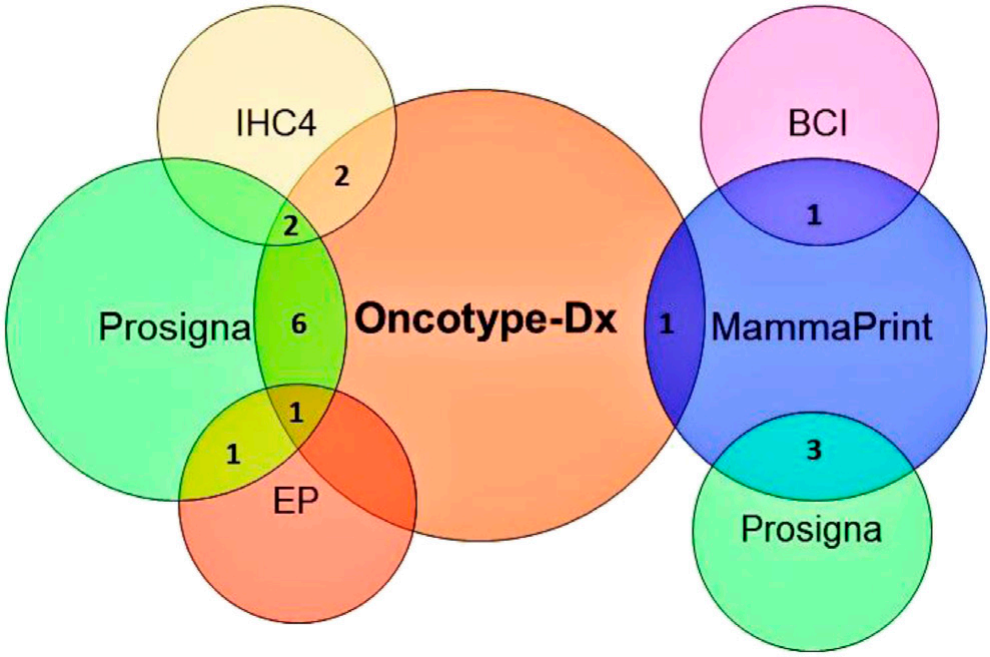
Venn diagram showing a shared number of genes/proteins between different multigene tests. BCI: breast cancer index, EP: endopredict, IHC4: immunohistochemical4.

Most of the multigene assays have overlapping target genes in their analysis (Figure 5). Though multigene prognostics are an effective means of detection, different prognostics’ results tell a different tale. A comparative study on 1) MammaPrint and Oncotype Dx RS (Nunes et al., 2016; Tsai et al., 2018), 2) Oncotype Dx and Prosigna (Dowsett et al., 2013), 3) TransATAC study on six different tests: EP, IHC4, Oncotype Dx, BCI, Prosigna, and clinical treatment score (that evaluates nodal status, tumor size, grade, age, and endocrine treatment beyond 5 years) (Sestak et al., 2018), observed different prognostics information in their patients. A discordance in findings reflects the importance of integrating additional factors such as clinicopathological information with genomic imprint analysis (Kwa et al., 2017).

## Conclusion and Future Perspectives

Despite large number of research articles published on BC diagnosis and treatment, research in its early detection still lags. Although several techniques have been developed in the last decade permitting detection and guidance on specific therapies, individual techniques’ pros and cons limit their utilization as a standalone tool. To plan a proper treatment, understanding the tumor’s molecular heterogeneity is crucial. An acute stratification is necessary as each group or sub-group exhibits individual prognosis and systemic therapy. Luminal A and B have an overlapping hormonal expression but have different prognosis and treatments. HER2^+^ BC has a better prognosis and shows a promising outcome upon merging chemotherapy with anti-HER2 monoclonal antibodies and tyrosine kinase inhibitor-based therapies. TNBC remains a challenge to treat. Recent studies have suggested that analyzing BC molecular subtypes can give better information on recurrence and treatment response.

The traditional classifications that include IHC-hormone evaluations, GEP analysis, and examining pathological features have become clinically more affordable in routine processes. However, gene transcription does not necessarily correlate with protein expression. Numerous factors, such as mRNA transcription rate, protein stability, post-translational modifications, and random mutations, also affect protein biomarkers’ therapeutic potential. Several genetic mutations predispose BC in women. Therefore, for a complete understanding of pathological changes in BC, a high-throughput analysis of data extracted from several “omics” studies is needed to interpret biomarkers and pathways involved.

The pursuit of suitable BC biomarkers took us in the era of big data or the era of “BC-Omics.” Genomics, metabolomics, and proteomics with predictive and prognostic BC biomarkers have laid the foundation of various multigene assays for early detection and treatment strategy. Currently, there are seven prognostic signature tests available for BC, MammaPrint, Breast Cancer Index, IHC4, EndoPredict, Prosigna, Mammostrat, and Oncotype Dx. Of the seven tests, Mammostrat and IHC4 are protein-based tests, while others are multi-gene-based screening. With multigene signature analysis alongside other molecular tools, clinicians can now select appropriate therapies and predict treatment duration more easily. A prognostic profile developed from multigene tests could better predict recurrence risk. Further, it could help avoid unnecessary treatments in low-risk patients so that they could resume their daily routines. As per ASCO guidelines, their use for screening BCs have not been approved. Clinical experts’ committees request more evidence to support multigene tests’ routine usage in HR^+^/HER2^−^ with node-positive BC in guiding adjuvant therapy. A comparative analysis of different multigene tests predicts distinct prognostics for the same patient, reflecting the importance of integrating clinicopathological information with genomic imprint analysis. Moreover, the cost of these tests is another obstacle for general use. Also, the expense of training the staff always remains an undervalued proposition that brings the challenge of maintaining the tests’ quality and reproducibility. Again, not all the tests are FDA approved. Additional clinical studies and results from large cohorts are needed to meet the ASCO requirements. There remains a gap between research findings and its clinical implementations. Several ongoing extensive cohort studies on BC, such as MINDACT, TAILORx, Trans-aTTOM, and many more, may provide positive impact on long-term patient outcome and guide the therapy.

Several clinical observations have found different treatment responses among diverse ethnic groups. In the era of personalized medicine, individualizing therapy is the next step in cancer treatment’s evolution. For a long time, human cell lines’ engraftment onto immunocompromized animals (Neve et al., 2006)—cell-derived xenografts (CDX)—have been used to understand BC genetics and its biological processes. With the advancement in understanding the importance of tumor microenvironment (Hanahan and Weinberg, 2011), the development of patient-derived xenografts (PDX) (Hoffman, 2015) circumvents the limitations with CDX. Unlike CDX, PDX models preserve the tumor’s heterogeneity, behavioral characteristics (metastasis), the microenvironment, and many other features. A unique advantage of PDX is its response to therapy (Tentler et al., 2012; Hidalgo et al., 2014). New models incorporate rare but aggressive BC (Wurth et al., 2015). Mimicking clinical trials of adjuvant therapy through experimental models such as PDX and syngeneic models will be significantly improved. Currently, these models of BC are amidst scientific debate in various immune-molecular societies (Varn et al., 2017) and are considered as mouse “avatars” for the patient (Holen et al., 2017). Besides, the immense amount of time and capital invested in such models’ development limits them to cohort-based preclinical studies. As such, a good *in vivo* model of BC is needed (Holen et al., 2017).

Furthermore, the discovery of the immune checkpoint proteins such as cytotoxic-T-lymphocyte-associated antigen-4 (CTLA-4) and Programmed Death-1 (PD-1) has led to a booming in immune-targeted therapies against tumors. Following targeted therapies’ strategy, oncolytic virus therapy (OVT) has shown potential in treating cancers, e.g., melanoma. Unlike gene therapy, where the virus is a carrier, OVT engineered the virus to target, infiltrate, multiply, and kill melanoma cells, leaving the normal cells unharmed. It includes four of the seven classes of viruses in the Baltimore classification system for BC treatment. Several scientific studies have obtained encouraging results with OVT based immune-targeted therapy. Therefore, mono-therapeutic approaches are rarely the best treatment option for BC. Collaboration across disciplines appears more promising and gaining traction in personalized treatments. With increasing knowledge and the advancement in diagnostics and treatment strategies, BC will become better understood and more manageable.

## GLOSSARY

AB, ABUS: Automated breast ultrasonography;
AI: Aromatase inhibitor
APCs: Antigen-presenting cells
AR: Androgen receptor
ASCO: American Society of Clinical Oncology
AT, ATM: Ataxia Telangiectasia, Ataxia Telangiectasia mutation
BC: Breast cancer(s)
BI-RADS: Breast imaging reporting and data system
BL: Basal-like
BLIA: Basal-like-immune-activated
BLIS: Basal-like-immune-suppressive
BRCA1/2: Breast cancer genes-1/-2
BRE: Biochemical recognition element
CA: Cancer antigen
cDNA: Complementary DNA
CDX: Cell-derived Xenografts
CES-MRI: Chemical exchange saturation transfer MRI
CEUS: Contrast-enhanced ultrasonography
CTCs: Circulating tumor cells
CTLA-4: Cytotoxic-T-lymphocyte-associated antigen
DBT: Digital breast tomosynthesis
DCE-MRI: Dynamic contrast-enhanced MRI
DCIS: Ductal carcinoma *in situ*
DIET: Digital image-based elasto-tomography
DITI: Digital infrared thermal imaging
EIS: Electrical impedance scanning/spectroscopy
ELISA: Enzyme-linked immunosorbent assay
EMT: Epithelial-mesenchymal transition
EP: EndoPredict
ER: Estrogen receptors
ER-α/PR: Estrogen and Progesterone receptor
ER^+^, ER^−^: ER-positive, ER-negative
ESMO: European society of medical oncology
GD-DTPA: Gadolinium diethylenetriamine pentaacetic acid
GEP: Gene expression profiling
HELU: Hyperplastic enlarged lobular unit
HER2: Human epidermal growth factor receptor 2
HER2-ECD: Extracellular domain of HER2
IARC: International Agency for Research on Cancer
IBC: Invasive breast carcinoma
IBC-NE: Invasive breast carcinoma with neuroendocrine differentiation
IDC: Invasive ductal carcinoma
IGF: Insulin-like growth factor
IHC: Immune histochemical/compatibility
ILC: Invasive lobular carcinoma
IM: Immunomodulatory
INSM1: Insulinoma-associated protein 1
LAR: Luminal androgen receptor
LncRNA: Long non-coding RNAs
LCIS: Lobular carcinoma *in situ*
M: Mitotic rate
MAPP: Multiple affinity protein profile
MGI: Molecular grade index
MES: Mesenchymal-like triple-negative breast cancer subtype
MMP: Matrix metalloproteinase
MMP-9: Matrix metalloproteinase-9
MRI: Magnetic resonance imaging
MSC: Mammary stem cell
MUC1: Mucin-1
N: Degree of nuclear polymorphism (Nodes)
NAAs: Nucleic acid aptamers
NCCN: American-national comprehensive cancer network
NE: Neuroendocrine
NET: Neuroendocrine tumor
NGS: Next-gene sequencing
NOS: Not otherwise specified
NPI: Nottingham prognosis Index
NSE: Neuron-specific enolase
NST: No special type
OVT: Oncolytic virus therapy
PD-1: Programmed death-1
PDX: Patient-derived xenografts
PR: Progesterone receptors
PTEN: Phosphatase and tensin homolog
PTM: Post-translational modification
QDs: Quantum dots
SELEX: Systemic evolution of ligands by exponential enrichment
SEREX: Serological analysis of tumor antigens by recombinant cDNA expression cloning
SERM: Selective estrogen receptor modulators
SERPA: Serological proteome assay
SIAH: Seven in absentia homolog
SPR: Surface plasmon resonance
T: Tubular structures
TAABs: Tumor-associated autoantibodies
TAAs: Tumor-associated antigens
TCCRP: Tall cell carcinoma with reverse polarity
TILs: Tumor-infiltrating lymphocytes
TNBC: Triple-negative breast cancer
WT: Wild type

## References

[B1] Alix-Panabières C. Pantel K. (2014). Challenges in circulating tumour cell research. Nat. Rev. Cancer 14 (9), 623–631. 10.1038/nrc3820 25154812

[B2] Alzurq E. Almaktari A. Aldin B. Hamoud M. Othman S. (2016). New system for early breast cancer detection by Electrical impedance spectroscopy. Recent Adv. Environ. Sci. Biomed. 4, 142–150.

[B3] Amioka A. Masumoto N. Gouda N. Kajitani K. Shigematsu H. Emi A. (2016). Ability of contrast-enhanced ultrasonography to determine clinical responses of breast cancer to neoadjuvant chemotherapy. Jpn. J. Clin. Oncol. 46 (4), 303–309. 10.1093/jjco/hyv215 26848078 PMC4886139

[B4] Anbar M. (1998). Clinical thermal imaging today. IEEE Eng. Med. Biol. Mag. 17 (4), 25–33. 10.1109/51.687960 9672807

[B5] Andre F. Ismaila N. Henry N. L. Somerfield M. R. Bast R. C. Barlow W. (2019). Use of biomarkers to guide decisions on adjuvant systemic therapy for women with early-stage invasive breast cancer: ASCO clinical practice guideline update-integration of results from TAILORx. J. Clin. Oncol. 37 (22), 1956–1964. 10.1200/JCO.19.00945 31150316

[B6] Anglian Breast Cancer Study Group (2000). Prevalence and penetrance of BRCA1 and BRCA2 mutations in a population-based series of breast cancer cases. Br. J. Cancer 83 (10), 1301. 10.1054/bjoc.2000.1407 11044354 PMC2408797

[B7] Arizti P. Fang L. Park I. Yin Y. Solomon E. Ouchi T. (2000). Tumor suppressor p53 is required to modulate BRCA1 expression. Mol. Cell Biol. 20 (20), 7450–7459. 10.1128/mcb.20.20.7450-7459.2000 11003642 PMC86298

[B8] Arkan E. Saber R. Karimi Z. Shamsipur M. (2015). A novel antibody-antigen based impedimetric immunosensor for low level detection of HER2 in serum samples of breast cancer patients via modification of a gold nanoparticles decorated multiwall carbon nanotube-ionic liquid electrode. Anal. Chim. Acta 874, 66–74. 10.1016/j.aca.2015.03.022 25910448

[B9] Arteaga C. L. Sliwkowski M. X. Osborne C. K. Perez E. A. Puglisi F. Gianni L. (2012). Treatment of HER2-positive breast cancer: current status and future perspectives. Nat. Rev. Clin. Oncol. 9 (1), 16–32. 10.1038/nrclinonc.2011.177 22124364

[B10] Arya S. K. Wang K. Y. Wong C. C. Rahman A. R. (2013). Anti-EpCAM modified LC-SPDP monolayer on gold microelectrode based electrochemical biosensor for MCF-7 cells detection. Biosens. Bioelectron. 41, 446–451. 10.1016/j.bios.2012.09.006 23021854

[B11] Atwood C. Hovey R. Glover J. Chepko G. Ginsburg E. Robison W. (2000). Progesterone induces side-branching of the ductal epithelium in the mammary glands of peripubertal mice. J. Endocrinol. 167 (1), 39–52. 10.1677/joe.0.1670039 11018751

[B12] Baccelli I. Schneeweiss A. Riethdorf S. Stenzinger A. Schillert A. Vogel V. (2013). Identification of a population of blood circulating tumor cells from breast cancer patients that initiates metastasis in a xenograft assay. Nat. Biotechnol. 31 (6), 539–544. 10.1038/nbt.2576 23609047

[B13] Barazi H. Gunduru M. (2019). Mammography BI RADS grading. StatPearls. Treasure Island, FL: StatPearls Publishing. 30969638

[B14] Bardou V. J. Arpino G. Elledge R. M. Osborne C. K. Clark G. M. (2003). Progesterone receptor status significantly improves outcome prediction over estrogen receptor status alone for adjuvant endocrine therapy in two large breast cancer databases. J. Clin. Oncol. 21 (10), 1973–1979. 10.1200/JCO.2003.09.099 12743151

[B15] Bartlett J. Sgroi D. C. Treuner K. Zhang Y. Piper T. Salunga R. C. (2020). HER2 status and prediction of extended endocrine benefit with breast cancer index (BCI) in HR+ patients in the adjuvant tamoxifen: to offer more?(aTTom) trial. J. Clin. Oncol. 38 (15_suppl), 522. 10.1200/JCO.2020.38.15_suppl.522 31504126 PMC6927322

[B16] Bartlett J. M. Thomas J. Ross D. T. Seitz R. S. Ring B. Z. Beck R. A. (2010). Mammostrat as a tool to stratify breast cancer patients at risk of recurrence during endocrine therapy. Breast Cancer Res. 12 (4), R47. 10.1186/bcr2604 20615243 PMC2949634

[B17] Begam N. Jamil K. Raju S. G. (2017). Promoter hypermethylation of the ATM gene as a novel biomarker for breast cancer. Asian Pac. J. Cancer Prev. APJCP 18 (11), 3003. 10.22034/APJCP.2017.18.11.3003 29172272 PMC5773784

[B18] Behling K. C. Tang A. Freydin B. Chervoneva I. Kadakia S. Schwartz G. F. (2011). Increased SIAH expression predicts ductal carcinoma *in situ* (DCIS) progression to invasive carcinoma. Breast Cancer Res. Treat. 129 (3), 717–724. 10.1007/s10549-010-1254-8 21088888 PMC3730842

[B19] Benvidi A. Dehghani Firouzabadi A. Dehghan Tezerjani M. Moshtaghiun S. M. Mazloum-Ardakani M. Ansarin A. (2015). A highly sensitive and selective electrochemical DNA biosensor to diagnose breast cancer. J. Electroanalytical Chem. 750, 57–64. 10.1016/j.jelechem.2015.05.002

[B20] Bertwistle D. Swift S. Marston N. J. Jackson L. E. Crossland S. Crompton M. R. (1997). Nuclear location and cell cycle regulation of the BRCA2 protein. Cancer Res. 57 (24), 5485–5488. 9407955

[B21] Blanco-Prieto S. Barcia-Castro L. Páez de la Cadena M. Rodríguez-Berrocal F. J. Vázquez-Iglesias L. Botana-Rial M. I. (2017). Relevance of matrix metalloproteases in non-small cell lung cancer diagnosis. BMC cancer 17 (1), 823. 10.1186/s12885-017-3842-z 29207990 PMC5718060

[B22] Blum J. L. Flynn P. J. Yothers G. Asmar L. Geyer C. E. Jr Jacobs S. A. (2017). Anthracyclines in early breast cancer: the ABC trials-USOR 06-090, NSABP B-46-I/USOR 07132, and NSABP B-49 (NRG oncology). J. Clin. Oncol. 35 (23), 2647. 10.1200/JCO.2016.71.4147 28398846 PMC5549453

[B23] Boriachek K. Islam M. N. Gopalan V. Lam A. K. Nguyen N. T. Shiddiky M. J. A. (2017). Quantum dot-based sensitive detection of disease specific exosome in serum. Analyst 142 (12), 2211–2219. 10.1039/c7an00672a 28534915

[B24] Bourdon J. C. Khoury M. P. Diot A. Baker L. Fernandes K. Aoubala M. (2011). p53 mutant breast cancer patients expressing p53γ have as good a prognosis as wild-type p53 breast cancer patients. Breast Cancer Res. 13 (1), R7. 10.1186/bcr2811 21251329 PMC3109573

[B25] Boyd N. F. Guo H. Martin L. J. Sun L. Stone J. Fishell E. (2007). Mammographic density and the risk and detection of breast cancer. N. Engl. J. Med. 356 (3), 227–236. 10.1056/NEJMoa062790 17229950

[B26] Brekelmans C. Seynaeve C. Menke-Pluymers M. Brüggenwirth H. Tilanus-Linthorst M. Bartels C. (2006). Survival and prognostic factors in BRCA1-associated breast cancer. Ann. Oncol. 17 (3), 391–400. 10.1093/annonc/mdj095 16322115

[B27] Brekelmans C. Tilanus-Linthorst M. Seynaeve C. vd Ouweland A. Menke-Pluymers M. B. Bartels C. C. (2007). Tumour characteristics, survival and prognostic factors of hereditary breast cancer from BRCA2-, BRCA1- and non-BRCA1/2 families as compared to sporadic breast cancer cases. Eur. J. Cancer 43 (5), 867–876. 10.1016/j.ejca.2006.12.009 17307353

[B28] Brem R. F. Tabár L. Duffy S. W. Inciardi M. F. Guingrich J. A. Hashimoto B. E. (2015). Assessing improvement in detection of breast cancer with three-dimensional automated breast US in women with dense breast tissue: the SomoInsight Study. Radiology 274 (3), 663–673. 10.1148/radiol.14132832 25329763

[B29] Brisken C. (2002). Hormonal control of alveolar development and its implications for breast carcinogenesis. J. Mammary Gland Biol. Neoplasia 7 (1), 39–48. 10.1023/a:1015718406329 12160085

[B30] Brown R. Hann C. Chase J. G. Ray L. (2007). Discrete color-based Euclidean-invariant signatures for feature tracking in a DIET breast cancer screening system. Med. Imaging 2007: Physiol. Funct. Struct. Med. Images, Int. Soc. Opt. Photon. 6511, 65110D. 10.1117/12.711815

[B31] Burstein M. D. Tsimelzon A. Poage G. M. Covington K. R. Contreras A. Fuqua S. A. (2015). Comprehensive genomic analysis identifies novel subtypes and targets of triple-negative breast cancer. Clin. Cancer Res. 21 (7), 1688–1698. 10.1158/1078-0432.CCR-14-0432 25208879 PMC4362882

[B32] Bussolati G. Badve S. (2012). “Carcinomas with neuroendocrine features,” in WHO Classification of Tumours of the Breast. Editors Lakhani S. R. Ellis I. O. Schnitt S. J. Tan P. H. van de Vijver M. J. (Lyon, France: IARC Press), 62–63.

[B33] Cai K. Xu H. N. Singh A. Moon L. Haris M. Reddy R. (2014). Breast cancer redox heterogeneity detectable with chemical exchange saturation transfer (CEST) MRI. Mol. Imaging Biol. 16 (5), 670–679. 10.1007/s11307-014-0739-y 24811957 PMC4251869

[B34] Cardoso F. van’t Veer L. J. Bogaerts J. Slaets L. Viale G. Delaloge S. (2016). 70-Gene signature as an aid to treatment decisions in early-stage breast cancer. N. Engl. J. Med. 375 (8), 717–729. 10.1056/NEJMoa1602253 27557300

[B35] Cardoso F. van’t Veer L. Poncet C. Lopes Cardozo J. Delaloge S. Pierga J.-Y. (2020). MINDACT: long-term results of the large prospective trial testing the 70-gene signature MammaPrint as guidance for adjuvant chemotherapy in breast cancer patients. Am. Soc. Clin. Oncol. 38, 506. 10.1200/JCO.2020.38.15_suppl.506

[B36] Casás-Selves M. DeGregori J. (2011, How cancer shapes evolution and how evolution shapes cancer. Evo. Edu. Outreach. 4 (4), 624–634. 10.1007/s12052-011-0373-y PMC3660034 23705033

[B37] Chambers J. P. Arulanandam B. P. Matta L. L. Weis A. Valdes J. J. (2008). Biosensor recognition elements. Curr. Issues Mol. Biol. 10, 1–12. 10.1007/978-0-387-75936-4_1 18525101

[B38] Chang R. F. Chen H. H. Chang Y. C. Huang C. S. Chen J. H. Lo C. M. (2016). Quantification of breast tumor heterogeneity for ER status, HER2 status, and TN molecular subtype evaluation on DCE-MRI. Magn. Reson. Imaging 34 (6), 809–819. 10.1016/j.mri.2016.03.001 26968141

[B39] Chang Y. F. Hung S. H. Lee Y. J. Chen R. C. Su L. C. Lai C. S. (2011). Discrimination of breast cancer by measuring prostate-specific antigen levels in women's serum. Anal. Chem. 83 (13), 5324–5328. 10.1021/ac200754x 21591802

[B40] Chauveau N. Hamzaoui L. Rochaix P. Rigaud B. Voigt J. Morucci J. (1999). *Ex vivo* discrimination between normal and pathological tissues in human breast surgical biopsies using bioimpedance spectroscopy. Ann. N. Y Acad. Sci. 873 (1), 42–50. 10.1111/j.1749-6632.1999.tb09447.x 10372148

[B41] Cheang M. C. U. Chia S. K. Voduc D. Gao D. Leung S. Snider J. (2009). Ki67 index, HER2 status, and prognosis of patients with luminal B breast cancer. JNCI: J. Natl. Cancer Inst. 101 (10), 736–750. 10.1093/jnci/djp082 19436038 PMC2684553

[B42] Chen L. Zhang J. He Y. Ding X. Y. (2018). Matrix metalloproteinase-9 expression of GCTSC in peripheral tissue and central tissue of GCTB. J. Cell Biochem. 119 (7), 5805–5812. 10.1002/jcb.26766 29600575

[B43] Chen Y. Farmer A. A. Chen C. F. Jones D. C. Chen P. L. Lee W. H. (1996). BRCA1 is a 220-kDa nuclear phosphoprotein that is expressed and phosphorylated in a cell cycle-dependent manner. Cancer Res. 56 (14), 3168–3172. 8764100

[B44] Cheng A. K. Su H. Wang Y. A. Yu H. Z. (2009). Aptamer-based detection of epithelial tumor marker mucin 1 with quantum dot-based fluorescence readout. Anal. Chem. 81 (15), 6130–6139. 10.1021/ac901223q 19572710

[B45] Cheng F. Wang Z. Huang Y. Duan Y. Wang X. (2015). Investigation of salivary free amino acid profile for early diagnosis of breast cancer with ultra performance liquid chromatography-mass spectrometry. Clin. Chim. Acta 447, 23–31. 10.1016/j.cca.2015.05.008 25987308

[B46] Chia K. O’Brien M. Brown M. Lim E. (2015). Targeting the androgen receptor in breast cancer. Curr. Oncol. Rep. 17 (2), 4. 10.1007/s11912-014-0427-8 25665553

[B47] Chun L. Kim S.-E. Cho M. Choe W.-s. Nam J. Lee D. W. Lee Y. (2013). Electrochemical detection of HER2 using single stranded DNA aptamer modified gold nanoparticles electrode. Sensors Actuators B: Chem. 186, 446–450. 10.1016/j.snb.2013.06.046

[B48] Clark L. Jr (1956). Monitor and control of blood and tissue oxygen tensions ASAIO J. 2 (1), 41–48.

[B49] Couch F. J. Hart S. N. Sharma P. Toland A. E. Wang X. Miron P. (2015). Inherited mutations in 17 breast cancer susceptibility genes among a large triple-negative breast cancer cohort unselected for family history of breast cancer. J. Clin. Oncol. 33 (4), 304. 10.1200/JCO.2014.57.1414 25452441 PMC4302212

[B50] Creighton C. J. Fu X. Hennessy B. T. Casa A. J. Zhang Y. Gonzalez-Angulo A. M. (2010). Proteomic and transcriptomic profiling reveals a link between the PI3K pathway and lower estrogen-receptor (ER) levels and activity in ER+ breast cancer. Breast Cancer Res. 12 (3), R40–R12. 10.1186/bcr2594 20569503 PMC2917035

[B51] Creighton C. J. Kent Osborne C. van de Vijver M. J. Foekens J. A. Klijn J. G. Horlings H. M. (2009). Molecular profiles of progesterone receptor loss in human breast tumors. Breast Cancer Res. Treat. 114 (2), 287–299. 10.1007/s10549-008-0017-2 18425577 PMC2635926

[B52] Cristofanilli M. Broglio K. R. Guarneri V. Jackson S. Fritsche H. A. Islam R. (2007). Circulating tumor cells in metastatic breast cancer: biologic staging beyond tumor burden. Clin. Breast Cancer 7 (6), 471–479. 10.3816/cbc.2007.n.004 17386124

[B53] Cui M. Wang Y. Wang H. Wu Y. Luo X. (2017). A label-free electrochemical DNA biosensor for breast cancer marker BRCA1 based on self-assembled antifouling peptide monolayer. Sensors Actuators B: Chem. 244, 742–749. 10.1016/j.snb.2017.01.060

[B54] Cui X. Schiff R. Arpino G. Osborne C. K. Lee A. V. (2005). Biology of progesterone receptor loss in breast cancer and its implications for endocrine therapy. J. Clin. Oncol. 23 (30), 7721–7735. 10.1200/JCO.2005.09.004 16234531

[B55] Culha M. Stokes D. L. Griffin G. D. Vo-Dinh T. (2004). Application of a miniature biochip using the molecular beacon probe in breast cancer gene BRCA1 detection. Biosens. Bioelectron. 19 (9), 1007–1012. 10.1016/j.bios.2003.09.006 15018955

[B56] Cuzick J. Dowsett M. Pineda S. Wale C. Salter J. Quinn E. (2011). Prognostic value of a combined estrogen receptor, progesterone receptor, Ki-67, and human epidermal growth factor receptor 2 immunohistochemical score and comparison with the Genomic Health recurrence score in early breast cancer. J. Clin. Oncol. 29 (32), 4273–4278. 10.1200/JCO.2010.31.2835 21990413

[B57] Darlix A. Lamy P. J. Lopez-Crapez E. Braccini A. L. Firmin N. Romieu G. (2016). Serum NSE, MMP-9 and HER2 extracellular domain are associated with brain metastases in metastatic breast cancer patients: predictive biomarkers for brain metastases? Int. J. Cancer 139 (10), 2299–2311. 10.1002/ijc.30290 27464303

[B58] Deng C.-X. (2006). BRCA1: cell cycle checkpoint, genetic instability, DNA damage response and cancer evolution. Nucleic Acids Res. 34 (5), 1416–1426. 10.1093/nar/gkl010 16522651 PMC1390683

[B59] Dey D. Goswami T. (2011). Optical biosensors: a revolution towards quantum nanoscale electronics device fabrication, J. Biomed. Biotechnol., 2011, 348218. 10.1155/2011/348218 22131802 PMC3205924

[B60] Dillenburg C. V. Bandeira I. C. Tubino T. V. Rossato L. G. Dias E. S. Bittelbrunn A. C. (2012). Prevalence of 185delAG and 5382insC mutations in BRCA1, and 6174delT in BRCA2 in women of Ashkenazi Jewish origin in southern Brazil. Genet. Mol. Biol. 35 (3), 599–602. 10.1590/S1415-47572012000400009 23055798 PMC3459409

[B61] Dowsett M. Houghton J. Iden C. Salter J. Farndon J. A’hern R. (2006). Benefit from adjuvant tamoxifen therapy in primary breast cancer patients according oestrogen receptor, progesterone receptor, EGF receptor and HER2 status. Ann. Oncol. 17 (5), 818–826. 10.1093/annonc/mdl016 16497822

[B62] Dowsett M. Sestak I. Lopez-Knowles E. Sidhu K. Dunbier A. K. Cowens J. W. (2013). Comparison of PAM50 risk of recurrence score with oncotype DX and IHC4 for predicting risk of distant recurrence after endocrine therapy. J. Clin. Oncol. 31 (22), 2783–2790. 10.1200/JCO.2012.46.1558 23816962

[B63] Dziembowska M. Wlodarczyk J. (2012). MMP9: a novel function in synaptic plasticity. Int. J. Biochem. Cell Biol. 44 (5), 709–713. 10.1016/j.biocel.2012.01.023 22326910

[B64] Eletxigerra U. Martinez-Perdiguero J. Merino S. Barderas R. Torrente-Rodríguez R. Villalonga R. (2015). Amperometric magnetoimmunosensor for ErbB2 breast cancer biomarker determination in human serum, cell lysates and intact breast cancer cells. Biosens. Bioelectron. 70, 34–41. 10.1016/j.bios.2015.03.017 25791465

[B65] Ellis I. Pinder S. Bobrow L. Buley I. Coyne J. Going J. (2005). Pathology reporting of breast disease. London, United Kingdom: NHS Publications.

[B66] Ellis M. J. Coop A. Singh B. Mauriac L. Llombert-Cussac A. Jänicke F. (2001). Letrozole is more effective neoadjuvant endocrine therapy than tamoxifen for ErbB-1- and/or ErbB-2-positive, estrogen receptor-positive primary breast cancer: evidence from a phase III randomized trial. J. Clin. Oncol. 19 (18), 3808–3816. 10.1200/JCO.2001.19.18.3808 11559718

[B67] Ellis M. J. Tao Y. Young O. White S. Proia A. D. Murray J. (2006). Estrogen-independent proliferation is present in estrogen-receptor HER2-positive primary breast cancer after neoadjuvant letrozole. J. Clin. Oncol. 24 (19), 3019–3025. 10.1200/JCO.2005.04.3034 16754938

[B68] Evans D. Howell A. Ward D. Lalloo F. Jones J. Eccles D. (2011). Prevalence of BRCA1 and BRCA2 mutations in triple negative breast cancer. J. Med. Genet. 48 (8), 520–522. 10.1136/jmedgenet-2011-100006 21653198

[B69] Felizi R. T. Veiga M. G. Carelli Filho I. Souto R. P. D. Fernandes C. E. Oliveira E. (2018). Association between matrix metallopeptidase 9 polymorphism and breast cancer risk. Rev. Bras Ginecol Obstet. 40 (10), 620–624. 10.1055/s-0038-1673366 30352460 PMC10316932

[B70] Feng L. Chen Y. Ren J. Qu X. (2011). A graphene functionalized electrochemical aptasensor for selective label-free detection of cancer cells. Biomaterials 32 (11), 2930–2937. 10.1016/j.biomaterials.2011.01.002 21256585

[B71] Filipits M. Rudas M. Jakesz R. Dubsky P. Fitzal F. Singer C. F. (2011). A new molecular predictor of distant recurrence in ER-positive, HER2-negative breast cancer adds independent information to conventional clinical risk factors. Clin. Cancer Res. 17 (18), 6012–6020. 10.1158/1078-0432.CCR-11-0926 21807638

[B72] Filipits M. Dubsky P. C. Rudas M. Brase J. C. Kronenwett R. Weber K. E. (2012). Impact of the EndoPredict-clin score on risk stratification in ER-positive, HER2-negative breast cancer after considering clinical guidelines, Am. Soc. Clin. Oncol. 30 (15), 542. 10.1200/jco.2012.30.15_suppl.542

[B73] Foulkes W. D. Stefansson I. M. Chappuis P. O. Bégin L. R. Goffin J. R. Wong N. (2003). Germline BRCA1 mutations and a basal epithelial phenotype in breast cancer. J. Natl. Cancer Inst. 95 (19), 1482–1485. 10.1093/jnci/djg050 14519755

[B74] Freitas M. Neves M. M. P. S. Nouws H. P. A. Delerue-Matos C. (2020). Quantum dots as nanolabels for breast cancer biomarker HER2-ECD analysis in human serum. Talanta 208, 120430. 10.1016/j.talanta.2019.120430 31816682

[B75] Fujita T. Doihara H. Kawasaki K. Takabatake D. Takahashi H. Washio K. (2006). PTEN activity could be a predictive marker of trastuzumab efficacy in the treatment of ErbB2-overexpressing breast cancer. Br. J. Cancer 94 (2), 247–252. 10.1038/sj.bjc.6602926 16404430 PMC2361109

[B76] Ganau S. Andreu F. J. Escribano F. Martín A. Tortajada L. Villajos M. (2015). Shear-wave elastography and immunohistochemical profiles in invasive breast cancer: evaluation of maximum and mean elasticity values. Eur. J. Radiol. 84 (4), 617–622. 10.1016/j.ejrad.2014.12.020 25619502

[B77] Gao J. Aksoy B. A. Dogrusoz U. Dresdner G. Gross B. Sumer S. O. (2013). Integrative analysis of complex cancer genomics and clinical profiles using the cBioPortal, Sci. Signal., 6, pl1. 10.1126/scisignal.2004088 23550210 PMC4160307

[B78] Gialeli C. Theocharis A. D. Karamanos N. K. (2011). Roles of matrix metalloproteinases in cancer progression and their pharmacological targeting. FEBS J. 278 (1), 16–27. 10.1111/j.1742-4658.2010.07919.x 21087457

[B79] Gibson G. R. Qian D. Ku J. K. Lai L. L. (2005). Metaplastic breast cancer: clinical features and outcomes. Am. Surg. 71 (9), 725–730. 10.1177/000313480507100906 16468506

[B80] Godet I. Gilkes D. M. (2017). BRCA1 and BRCA2 mutations and treatment strategies for breast cancer. Integr. Cancer Sci. Ther. 4. 10.15761/ICST.1000228 PMC5505673 28706734

[B81] Golubnitschaja O. Yeghiazaryan K. Abraham J. A. Schild H. H. Costigliola V. Debald M. (2017). Breast cancer risk assessment: a non-invasive multiparametric approach to stratify patients by MMP-9 serum activity and RhoA expression patterns in circulating leucocytes. Amino Acids 49 (2), 273–281. 10.1007/s00726-016-2357-2 27812894

[B82] Goto Y. De Silva M. Toscani A. Prabhakar B. Notkins A. Lan M. (1992). A novel human insulinoma-associated cDNA, IA-1, encodes a protein with “zinc-finger” DNA-binding motifs. J. Biol. Chem. 267 (21), 15252–15257. 10.1016/s0021-9258(18)42173-4 1634555

[B83] Grieshaber D. MacKenzie R. Vörös J. Reimhult E. (2008). Electrochemical biosensors—sensor principles and architectures. Sensors (Basel) 8 (3), 1400–1458. 10.3390/s80314000 27879772 PMC3663003

[B84] Haeri Z. Shokoufi M. Jenab M. Janzen R. Golnaraghi F. (2016). Electrical impedance spectroscopy for breast cancer diagnosis: clinical study. Integr. Cancer Sci. Ther. 3 (6), 1–6. 10.15761/icst.1000212

[B85] Hamrita B. Chahed K. Kabbage M. Guillier C. L. Trimeche M. Chaïeb A. (2008). Identification of tumor antigens that elicit a humoral immune response in breast cancer patients’ sera by serological proteome analysis (SERPA). Clin. Chim. Acta 393 (2), 95–102. 10.1016/j.cca.2008.03.017 18424265

[B86] Hanahan D. Weinberg R. A. (2011). Hallmarks of cancer: the next generation. Cell 144 (5), 646–674. 10.1016/j.cell.2011.02.013 21376230

[B87] Harris L. N. Ismaila N. McShane L. M. Andre F. Collyar D. E. Gonzalez-Angulo A. M. (2016). Use of biomarkers to guide decisions on adjuvant systemic therapy for women with early-stage invasive breast cancer: American Society of Clinical Oncology Clinical Practice Guideline. J. Clin. Oncol. 34 (10), 1134. 10.1200/JCO.2015.65.2289 26858339 PMC4933134

[B88] Hauge I. H. Pedersen K. Olerud H. M. Hole E. O. Hofvind S. (2014). The risk of radiation-induced breast cancers due to biennial mammographic screening in women aged 50–69 years is minimal. Acta Radiol. 55 (10), 1174–1179. 10.1177/0284185113514051 24311702

[B89] Haurum J. S. (2006). Recombinant polyclonal antibodies: the next generation of antibody therapeutics? Drug Discov. Today 11 (13-14), 655–660. 10.1016/j.drudis.2006.05.009 16793535

[B90] Hayes D. F. Thor A. D. Dressler L. G. Weaver D. Edgerton S. Cowan D. (2007). HER2 and response to paclitaxel in node-positive breast cancer. N. Engl. J. Med. 357 (15), 1496–1506. 10.1056/NEJMoa071167 17928597

[B91] He Y. Lin Y. Tang H. Pang D. (2012). A graphene oxide-based fluorescent aptasensor for the turn-on detection of epithelial tumor marker mucin 1. Nanoscale 4 (6), 2054–2059. 10.1039/c2nr12061e 22336777

[B92] Hidalgo M. Amant F. Biankin A. V. Budinská E. Byrne A. T. Caldas C. (2014). Patient-derived xenograft models: an emerging platform for translational cancer research. Cancer Discov. 4 (9), 998–1013. 10.1158/2159-8290.CD-14-0001 25185190 PMC4167608

[B93] Hoffman R. M. (2015). Patient-derived orthotopic xenografts: better mimic of metastasis than subcutaneous xenografts. Nat. Rev. Cancer 15 (8), 451–452. 10.1038/nrc3972 26422835

[B94] Holen I. Speirs V. Morrissey B. Blyth K. (2017). *In vivo* models in breast cancer research: progress, challenges and future directions. Dis. Model Mech. 10 (4), 359–371. 10.1242/dmm.028274 28381598 PMC5399571

[B95] Hoon Tan P. Ellis I. Allison K. Brogi E. Fox S. B. Lakhani S. (2020). The 2019 WHO classification of tumours of the breast. Histopathology 77 (2), 181–185. 10.1111/his.14091 32056259

[B96] Hu R. Wen W. Wang Q. Xiong H. Zhang X. Gu H. (2014). Novel electrochemical aptamer biosensor based on an enzyme-gold nanoparticle dual label for the ultrasensitive detection of epithelial tumour marker MUC1. Biosens. Bioelectron. 53, 384–389. 10.1016/j.bios.2013.10.015 24189297

[B97] Hu Z. Fan C. Oh D. S. Marron J. He X. Qaqish B. F. (2006). The molecular portraits of breast tumors are conserved across microarray platforms. BMC genomics 7 (1), 96–12. 10.1186/1471-2164-7-96 16643655 PMC1468408

[B98] Hubbard R. A. Kerlikowske K. Flowers C. I. Yankaskas B. C. Zhu W. Miglioretti D. L. (2011). Cumulative probability of false-positive recall or biopsy recommendation after 10 years of screening mammography: a cohort study. Ann. Intern. Med. 155 (8), 481–492. 10.7326/0003-4819-155-8-201110180-00004 22007042 PMC3209800

[B99] Hwang L. A. Phang B. H. Liew O. W. Iqbal J. Koh X. H. Koh X. Y. (2018). Monoclonal antibodies against specific p53 hotspot mutants as potential tools for precision medicine. Cell Rep 22 (1), 299–312. 10.1016/j.celrep.2017.11.112 29298430

[B100] Jin L. Chen K. Tan C. Li J. Luo J. Yang Y. (2020). Prognostic value of modified IHC4 score in patients with estrogen receptor–positive metastatic breast cancer. Oncologist 25 (8), e1170–e1180. 10.1634/theoncologist.2019-1006 32476192 PMC7418366

[B101] Jossinet J. (1996). Variability of impedivity in normal and pathological breast tissue. Med. Biol. Eng. Comput. 34 (5), 346–350. 10.1007/BF02520002 8945857

[B102] Karami F. Mehdipour P. (2013). A comprehensive focus on global spectrum of BRCA1 and BRCA2 mutations in breast cancer. Biomed. Res. Int., 2013, 928562. 10.1155/2013/928562 24312913 PMC3838820

[B103] Kessenbrock K. Plaks V. Werb Z. (2010). Matrix metalloproteinases: regulators of the tumor microenvironment. Cell 141 (1), 52–67. 10.1016/j.cell.2010.03.015 20371345 PMC2862057

[B104] Kim B. K. Lee J. W. Park P. J. Shin Y. S. Lee W. Y. Lee K. A. (2009). The multiplex bead array approach to identifying serum biomarkers associated with breast cancer. Breast Cancer Res. 11 (2), R22. 10.1186/bcr2247 19400944 PMC2688951

[B105] Kim S. An S. S. (2016). Role of p53 isoforms and aggregations in cancer, Medicine (Baltimore), 95, e3993. 10.1097/MD.0000000000003993 27368003 PMC4937917

[B106] King M. C. Marks J. H. Mandell J. B. (2003). Breast and ovarian cancer risks due to inherited mutations in BRCA1 and BRCA2. Science 302 (5645), 643–646. 10.1126/science.1088759 14576434

[B107] Kolb T. M. Lichy J. Newhouse J. H. (2002). Comparison of the performance of screening mammography, physical examination, and breast US and evaluation of factors that influence them: an analysis of 27,825 patient evaluations. Radiology 225 (1), 165–175. 10.1148/radiol.2251011667 12355001

[B108] Korsching E. Packeisen J. Agelopoulos K. Eisenacher M. Voss R. Isola J. (2002). Cytogenetic alterations and cytokeratin expression patterns in breast cancer: integrating a new model of breast differentiation into cytogenetic pathways of breast carcinogenesis. Lab. Invest. 82 (11), 1525–1533. 10.1097/01.lab.0000038508.86221.b3 12429812

[B109] Kouros-Mehr H. Slorach E. M. Sternlicht M. D. Werb Z. (2006). GATA-3 maintains the differentiation of the luminal cell fate in the mammary gland. Cell 127 (5), 1041–1055. 10.1016/j.cell.2006.09.048 17129787 PMC2646406

[B110] Kronenwett U. Ploner A. Zetterberg A. Bergh J. Hall P. Auer G. (2006). Genomic instability and prognosis in breast carcinomas. Cancer Epidemiol. Biomarkers Prev. 15 (9), 1630–1635. 10.1158/1055-9965.EPI-06-0080 16985023

[B111] Krop I. Ismaila N. Andre F. Bast R. C. Barlow W. Collyar D. E. (2017, Use of biomarkers to guide decisions on adjuvant systemic therapy for women with early-stage invasive breast cancer: American society of clinical oncology clinical practice guideline focused update). Jco 35 (24), 2838. 10.1200/jco.2017.74.0472 PMC5846188 28692382

[B112] Kwa M. Makris A. Esteva F. J. (2017). Clinical utility of gene-expression signatures in early stage breast cancer. Nat. Rev. Clin. Oncol. 14 (10), 595–610. 10.1038/nrclinonc.2017.74 28561071

[B113] Labrie F. Luu-The V. Labrie C. Bélanger A. Simard J. Lin S. X. (2003). Endocrine and intracrine sources of androgens in women: inhibition of breast cancer and other roles of androgens and their precursor dehydroepiandrosterone. Endocr. Rev. 24 (2), 152–182. 10.1210/er.2001-0031 12700178

[B114] Laidi F. Bouziane A. Errachid A. Zaoui F. (2016). Usefulness of salivary and serum auto-antibodies against tumor biomarkers HER2 and MUC1 in breast cancer screening. Asian Pac. J. Cancer Prev. 17 (1), 335–339. 10.7314/apjcp.2016.17.1.335 26838233

[B115] Lakhani S. R. Ellis I. O. Schnitt S. Tan P. H. van de Vijver M. (2012). WHO classification of tumours of the breast. Lyon, France: International Agency for Research on Cancer.

[B116] Lee A. H. Ellis I. O. (2008). The Nottingham prognostic index for invasive carcinoma of the breast. Pathol. Oncol. Res. 14 (2), 113–115. 10.1007/s12253-008-9067-3 18543079

[B117] Lee S. Mohsin S. K. Mao S. Hilsenbeck S. G. Medina D. Allred D. C. (2005). Hormones, receptors, and growth in hyperplastic enlarged lobular units: early potential precursors of breast cancer. Breast Cancer Res. 8 (1), R6–R9. 10.1186/bcr1367 16417654 PMC1413990

[B118] Lee S. H. Moon W. K. Cho N. Chang J. M. Moon H. G. Han W. (2014). Shear-wave elastographic features of breast cancers: comparison with mechanical elasticity and histopathologic characteristics. Invest. Radiol. 49 (3), 147–155. 10.1097/RLI.0000000000000006 24169069

[B119] Lehmann B. D. Bauer J. A. Chen X. Sanders M. E. Chakravarthy A. B. Shyr Y. (2011). Identification of human triple-negative breast cancer subtypes and preclinical models for selection of targeted therapies. J. Clin. Invest. 121 (7), 2750–2767. 10.1172/JCI45014 21633166 PMC3127435

[B120] Li D. Cheng W. Yan Y. Zhang Y. Yin Y. Ju H. (2016). A colorimetric biosensor for detection of attomolar microRNA with a functional nucleic acid-based amplification machine. Talanta 146, 470–476. 10.1016/j.talanta.2015.09.010 26695292

[B121] Li L. N. Zhou X. Gu Y. Yan J. (2013). Prognostic value of MMP-9 in ovarian cancer: a meta-analysis. Asian Pac. J. Cancer Prev. 14 (7), 4107–4113. 10.7314/apjcp.2013.14.7.4107 23991961

[B122] Li Y. Wu T. Zhang B. Yao Y. Yin G. (2012). Matrix metalloproteinase-9 is a prognostic marker for patients with cervical cancer. Med. Oncol. 29 (5), 3394–3399. 10.1007/s12032-012-0283-z 22752570

[B123] Li Y. Zhang Y. Zhao M. Zhou Q. Wang L. Wang H. (2016). A simple aptamer-functionalized gold nanorods based biosensor for the sensitive detection of MCF-7 breast cancer cells. Chem. Commun. (Camb) 52 (20), 3959–3961. 10.1039/c6cc01014h 26882343

[B124] Li Y. J. Wen G. Wang Y. Wang D. X. Yang L. Deng Y. J. (2013). Perfusion heterogeneity in breast tumors for assessment of angiogenesis. J. Ultrasound Med. 32 (7), 1145–1155. 10.7863/ultra.32.7.1145 23804337

[B125] Liang Y. H. Chang C. C. Chen C. C. Chu-Su Y. Lin C. W. (2012). Development of an Au/ZnO thin film surface plasmon resonance-based biosensor immunoassay for the detection of carbohydrate antigen 15-3 in human saliva. Clin. Biochem. 45 (18), 1689–1693. 10.1016/j.clinbiochem.2012.09.001 22981930

[B126] Liu S. Dontu G. Wicha M. S. (2005). Mammary stem cells, self-renewal pathways, and carcinogenesis. Breast Cancer Res. 7 (3), 86. 10.1186/bcr1021 15987436 PMC1143566

[B127] Liu Y. R. Jiang Y. Z. Xu X. E. Yu K. D. Jin X. Hu X. (2016). Comprehensive transcriptome analysis identifies novel molecular subtypes and subtype-specific RNAs of triple-negative breast cancer. Breast Cancer Res. 18 (1), 33. 10.1186/s13058-016-0690-8 26975198 PMC4791797

[B128] Marabelli M. Cheng S. C. Parmigiani G. (2016). Penetrance of ATM gene mutations in breast cancer: a meta-analysis of different measures of risk. Genet. Epidemiol. 40 (5), 425–431. 10.1002/gepi.21971 27112364 PMC7376952

[B129] Marcel V. Dichtel-Danjoy M. L. Sagne C. Hafsi H. Ma D. Ortiz-Cuaran S. (2011). Biological functions of p53 isoforms through evolution: lessons from animal and cellular models. Cell Death Differ 18 (12), 1815–1824. 10.1038/cdd.2011.120 21941372 PMC3214904

[B130] Marques R. C. Viswanathan S. Nouws H. P. Delerue-Matos C. González-García M. B. (2014). Electrochemical immunosensor for the analysis of the breast cancer biomarker HER2 ECD. Talanta 129, 594–599. 10.1016/j.talanta.2014.06.035 25127638

[B131] Martin M. Brase J. C. Calvo L. Krappmann K. Ruiz-Borrego M. Fisch K. (2014). Clinical validation of the EndoPredict test in node-positive, chemotherapy-treated ER+/HER2- breast cancer patients: results from the GEICAM 9906 trial. Breast Cancer Res. 16 (2), R38. 10.1186/bcr3642 24725534 PMC4076639

[B132] Mavaddat N. Peock S. Frost D. Ellis S. Platte R. Fineberg E. (2013, Cancer risks for BRCA1 and BRCA2 mutation carriers: results from prospective analysis of EMBRACE. JNCI: J. Natl. Cancer Inst. 105 (11), 812–822. 10.1093/jnci/djt095 23628597

[B133] Medhioub M. Vaury C. Hamelin R. Thomas G. (2000). Lack of somatic mutation in the coding sequence of SIAH1 in tumors hemizygous for this candidate tumor suppressor gene. Int. J. Cancer 87 (6), 794–797. 10.1002/1097-0215(20000915)87:6<794::aid-ijc5>3.0.co;2-b 10956387

[B134] Mehner C. Hockla A. Miller E. Ran S. Radisky D. C. Radisky E. S. (2014). Tumor cell-produced matrix metalloproteinase 9 (MMP-9) drives malignant progression and metastasis of basal-like triple negative breast cancer. Oncotarget 5 (9), 2736. 10.18632/oncotarget.1932 24811362 PMC4058041

[B135] Milella M. Falcone I. Conciatori F. Cesta Incani U. Del Curatolo A. Inzerilli N. (2015). PTEN: multiple functions in human malignant tumors. Front. Oncol. 5, 24. 10.3389/fonc.2015.00024 25763354 PMC4329810

[B136] Morimoto T. Kinouchi Y. Iritani T. Kimura S. Konishi Y. Mitsuyama N. (1990). Measurement of the electrical bio-impedance of breast tumors. Eur. Surg. Res. 22 (2), 86–92. 10.1159/000129087 2384126

[B137] Moy L. Elias K. Patel V. Lee J. Babb J. S. Toth H. K. (2009). Is breast MRI helpful in the evaluation of inconclusive mammographic findings? AJR Am. J. Roentgenol 193 (4), 986–993. 10.2214/AJR.08.1229 19770320

[B138] Mrklić I. Pogorelić Z. Capkun V. Tomić S. (2013). Expression of androgen receptors in triple negative breast carcinomas. Acta Histochem. 115 (4), 344–348. 10.1016/j.acthis.2012.09.006 23031358

[B139] Musgrove E. A. Sutherland R. L. (2009). Biological determinants of endocrine resistance in breast cancer. Nat. Rev. Cancer 9 (9), 631–643. 10.1038/nrc2713 19701242

[B140] Musolino A. Bella M. A. Bortesi B. Michiara M. Naldi N. Zanelli P. (2007). BRCA mutations, molecular markers, and clinical variables in early-onset breast cancer: a population-based study. Breast 16 (3), 280–292. 10.1016/j.breast.2006.12.003 17257844

[B141] Neo S. J. Su X. Thomsen J. S. (2009). Surface plasmon resonance study of cooperative interactions of estrogen receptor alpha and transcriptional factor Sp1 with composite DNA elements. Anal. Chem. 81 (9), 3344–3349. 10.1021/ac802543x 19331400

[B142] Network C. G. A. (2012). Comprehensive molecular portraits of human breast tumours. Nature 490 (7418), 61. 10.1038/nature11412 23000897 PMC3465532

[B143] Neve R. M. Chin K. Fridlyand J. Yeh J. Baehner F. L. Fevr T. (2006). A collection of breast cancer cell lines for the study of functionally distinct cancer subtypes. Cancer Cell 10 (6), 515–527. 10.1016/j.ccr.2006.10.008 17157791 PMC2730521

[B144] Nielsen T. Wallden B. Schaper C. Ferree S. Liu S. Gao D. (2014). Analytical validation of the PAM50-based Prosigna breast cancer prognostic gene signature assay and nCounter analysis system using formalin-fixed paraffin-embedded breast tumor specimens. BMC Cancer 14 (1), 177. 10.1186/1471-2407-14-177 24625003 PMC4008304

[B145] Nikhil B. Pawan J. Nello F. Pedro E. (2016). Introduction to biosensors. Essays Biochem. 60 (1), 1–8. 10.1042/EBC20150001 27365030 PMC4986445

[B146] Nuciforo P. G. Aura C. Holmes E. Prudkin L. Jimenez J. Martinez P. (2015). Benefit to neoadjuvant anti-human epidermal growth factor receptor 2 (HER2)-targeted therapies in HER2-positive primary breast cancer is independent of phosphatase and tensin homolog deleted from chromosome 10 (PTEN) status. Ann. Oncol. 26 (7), 1494–1500. 10.1093/annonc/mdv175 25851628 PMC5006510

[B147] Nunes R. A. Wray L. Mete M. Herbolsheimer P. Smith K. L. Bijelic L. (2016). Genomic profiling of breast cancer in African-American women using MammaPrint. Breast Cancer Res. Treat. 159 (3), 481–488. 10.1007/s10549-016-3949-y 27568021

[B148] Ollmar S. Grant S. (2016). Nevisense: improving the accuracy of diagnosing melanoma. Melanoma Manag. 3 (2), 93–96. 10.2217/mmt-2015-0004 30190877 PMC6094649

[B149] Oyama T. Takei K. Horiguchi H. Nakajima J. Koerner Y. Nakajima T. (2000). Atypical cystic lobule of the breast: an early stage of low-grade ductal carcinoma *in-situ* . Breast Cancer 7 (4), 326–331. 10.1007/BF02966399 11114859

[B150] Ozturk L. K. Emekli-Alturfan E. Kaşikci E. Demir G. Yarat A. (2011). Salivary total sialic acid levels increase in breast cancer patients: a preliminary study. Med. Chem. 7 (5), 443–447. 10.2174/157340611796799230 21801151

[B151] Paik S. Shak S. Tang G. Kim C. Baker J. Cronin M. (2004). A multigene assay to predict recurrence of tamoxifen-treated, node-negative breast cancer. N. Engl. J. Med. 351 (27), 2817–2826. 10.1056/NEJMoa041588 15591335

[B152] Parker J. S. Mullins M. Cheang M. C. Leung S. Voduc D. Vickery T. (2009). Supervised risk predictor of breast cancer based on intrinsic subtypes. J. Clin. Oncol. 27 (8), 1160. 10.1200/JCO.2008.18.1370 19204204 PMC2667820

[B153] Perou C. M. Sørlie T. Eisen M. B. Van De Rijn M. Jeffrey S. S. Rees C. A. (2000). Molecular portraits of human breast tumours. nature 406 (6797), 747–752. 10.1038/35021093 10963602

[B154] Peters A. Chase J. G. Van Houten E. E. (2008). Digital image elasto-tomography: combinatorial and hybrid optimization algorithms for shape-based elastic property reconstruction. IEEE Trans. Biomed. Eng. 55 (11), 2575–2583. 10.1109/TBME.2008.2001132 18990627

[B155] Peters A. Chase J. G. Van Houten E. E. (2009). Estimating elasticity in heterogeneous phantoms using Digital Image Elasto-Tomography. Med. Biol. Eng. Comput. 47 (1), 67–76. 10.1007/s11517-008-0368-1 18931869

[B156] Pfeifer M. E. (2018). Quo vadis point-of-care diagnostics? Report II of the SWISS SYMPOSIUM in point-of-care diagnostics 2017. CHIMIA Int. J. Chem. 72 (1-2), 80–82. 10.2533/chimia.2018.80 29490803

[B157] Piccart-Gebhart M. J. Procter M. Leyland-Jones B. Goldhirsch A. Untch M. Smith I. (2005). Trastuzumab after adjuvant chemotherapy in HER2-positive breast cancer. N. Engl. J. Med. 353 (16), 1659–1672. 10.1056/NEJMoa052306 16236737

[B158] Pohanka M. (2018). Overview of piezoelectric biosensors, immunosensors and DNA sensors and their applications. Materials 11 (3), 448. 10.3390/ma11030448 PMC5873027 29562700

[B159] Prat A. Perou C. M. (2009). Mammary development meets cancer genomics. Nat. Med. 15 (8), 842–844. 10.1038/nm0809-842 19661985

[B160] Prevention C. (2020). The BRCA1 and BRCA2 genes. Available at: https://www.cdc.gov/genomics/disease/breast_ovarian_cancer/genes_hboc.htm (Accessed March 25, 2020).

[B161] Qureshi A. Gurbuz Y. Niazi J. H. (2015), Label-free capacitance based aptasensor platform for the detection of HER2/ErbB2 cancer biomarker in serum. Sensors Actuators B: Chem. 220, 1145–1151. 10.1016/j.snb.2015.06.094

[B162] Rahbar H. Partridge S. C. (2016). Multiparametric MR imaging of breast cancer. Magn. Reson. Imaging Clin. N. Am. 24 (1), 223–238. 10.1016/j.mric.2015.08.012 26613883 PMC4672390

[B163] Rahim B. O’Regan R. (2017). AR signaling in breast cancer. Cancers 9 (3), 21. 10.3390/cancers9030021 PMC5366816 28245550

[B164] Rakha E. A. El-Sayed M. E. Green A. R. Paish E. C. Powe D. G. Gee J. (2007). Biologic and clinical characteristics of breast cancer with single hormone receptor positive phenotype. J. Clin. Oncol. 25 (30), 4772–4778. 10.1200/JCO.2007.12.2747 17876012

[B165] Rakha E. A. Reis-Filho J. S. Ellis I. O. (2010). Combinatorial biomarker expression in breast cancer. Breast Cancer Res. Treat. 120 (2), 293–308. 10.1007/s10549-010-0746-x 20107892

[B166] Razvi H. Tsang J. Y. Poon I. K. Chan S.-K. Cheung S.-Y. Shea K.-H. (2020). INSM1 is a novel prognostic neuroendocrine marker for luminal B breast cancer. Pathology 53 (2), 170–178. 10.1016/j.pathol.2020.07.004 32951906

[B167] Reporting B. I. (2003). Data system Atlas (BI-RADS atlas) reston. Reston, VA: American College of Radiology.

[B168] Ring B. Z. Seitz R. S. Beck R. Shasteen W. J. Tarr S. M. Cheang M. C. (2006). Novel prognostic immunohistochemical biomarker panel for estrogen receptor-positive breast cancer. J. Clin. Oncol. 24 (19), 3039–3047. 10.1200/JCO.2006.05.6564 16809728

[B169] Rong Y. Chen H. Zhou X. F. Yin C. Q. Wang B. C. Peng C. W. (2016). Identification of an aptamer through whole cell-SELEX for targeting high metastatic liver cancers. Oncotarget 7 (7), 8282. 10.18632/oncotarget.6988 26882565 PMC4884992

[B170] Ruan W. Monaco M. E. Kleinberg D. L. (2005). Progesterone stimulates mammary gland ductal morphogenesis by synergizing with and enhancing insulin-like growth factor-I action. Endocrinology 146 (3), 1170–1178. 10.1210/en.2004-1360 15604210

[B171] Safarpour D. Pakneshan S. Tavassoli F. A. (2014). Androgen receptor (AR) expression in 400 breast carcinomas: is routine AR assessment justified? Am. J. Cancer Res. 4 (4), 353. 25057438 PMC4106653

[B172] Salgado R. Denkert C. Demaria S. Sirtaine N. Klauschen F. Pruneri G. (2015). The evaluation of tumor-infiltrating lymphocytes (TILs) in breast cancer: recommendations by an International TILs Working Group 2014. Ann. Oncol. 26 (2), 259–271. 10.1093/annonc/mdu450 25214542 PMC6267863

[B173] Schiff R. Massarweh S. A. Shou J. Bharwani L. Mohsin S. K. Osborne C. K. (2004). Cross-talk between estrogen receptor and growth factor pathways as a molecular target for overcoming endocrine resistance. Clin. Cancer Res. 10 (1), 331S. 10.1158/1078-0432.ccr-031212 14734488

[B174] Schmidt R. L. Park C. H. Ahmed A. U. Gundelach J. H. Reed N. R. Cheng S. (2007). Inhibition of RAS-mediated transformation and tumorigenesis by targeting the downstream E3 ubiquitin ligase seven in absentia homologue. Cancer Res. 67 (24), 11798–11810. 10.1158/0008-5472.CAN-06-4471 18089810

[B175] Sestak I. Buus R. Cuzick J. Dubsky P. Kronenwett R. Denkert C. (2018). Comparison of the performance of 6 prognostic signatures for estrogen receptor-positive breast cancer: a secondary analysis of a randomized clinical trial. JAMA Oncol. 4 (4), 545–553. 10.1001/jamaoncol.2017.5524 29450494 PMC5885222

[B176] Sgroi D. C. Sestak I. Cuzick J. Zhang Y. Schnabel C. A. Schroeder B. (2013). Prediction of late distant recurrence in patients with oestrogen-receptor-positive breast cancer: a prospective comparison of the breast-cancer index (BCI) assay, 21-gene recurrence score, and IHC4 in the TransATAC study population. Lancet Oncol. 14 (11), 1067–1076. 10.1016/S1470-2045(13)70387-5 24035531 PMC3918681

[B177] Shackleton M. Vaillant F. Simpson K. J. Stingl J. Smyth G. K. Asselin-Labat M. L. (2006). Generation of a functional mammary gland from a single stem cell. Nature 439 (7072), 84–88. 10.1038/nature04372 16397499

[B178] Shah S. P. Roth A. Goya R. Oloumi A. Ha G. Zhao Y. (2012). The clonal and mutational evolution spectrum of primary triple-negative breast cancers. Nature 486 (7403), 395–399. 10.1038/nature10933 22495314 PMC3863681

[B179] Shao M. M. Chan S. K. Yu A. M. Lam C. C. Tsang J. Y. Lui P. C. (2012). Keratin expression in breast cancers. Virchows Arch. 461 (3), 313–322. 10.1007/s00428-012-1289-9 22851038

[B180] Sharan S. K. Morimatsu M. Albrecht U. Lim D. S. Regel E. Dinh C. (1997). Embryonic lethality and radiation hypersensitivity mediated by Rad51 in mice lacking Brca2. Nature 386 (6627), 804–810. 10.1038/386804a0 9126738

[B181] Shi H. Wu Y. Wang Y. Zhou M. Yan S. Chen Z. (2015). Liquiritigenin potentiates the inhibitory effects of cisplatin on invasion and metastasis via downregulation MMP-2/9 and PI3 K/AKT signaling pathway in B16F10 melanoma cells and mice model. Nutr. Cancer 67 (5), 761–770. 10.1080/01635581.2015.1037962 25978595

[B182] Siegel R. Jemal A. (2015). Cancer facts and figures 2015. Atlanta, GA: American Cancer Society Inc.

[B183] Singh A. K. Yu X. (2020). Tissue-Specific carcinogens as soil to seed BRCA1/2-mutant hereditary cancers. Trends Cancer 6 (7), 559–568. 10.1016/j.trecan.2020.03.004 32336659

[B184] Sinn H. P. Kreipe H. (2013). A brief overview of the WHO classification of breast tumors, 4th edition, focusing on issues and updates from the 3rd edition. Breast Care 8 (2), 149–154. 10.1159/000350774 24415964 PMC3683948

[B185] Slamon D. J. Godolphin W. Jones L. A. Holt J. A. Wong S. G. Keith D. E. (1989). Studies of the HER-2/neu proto-oncogene in human breast and ovarian cancer. Science 244 (4905), 707–712. 10.1126/science.2470152 2470152

[B186] Smerage J. B. Budd G. T. Doyle G. V. Brown M. Paoletti C. Muniz M. (2013). Monitoring apoptosis and Bcl-2 on circulating tumor cells in patients with metastatic breast cancer. Mol. Oncol. 7 (3), 680–692. 10.1016/j.molonc.2013.02.013 23538216 PMC5528485

[B187] Smith S. Easton D. Evans D. Ponder B. (1992). Allele losses in the region 17q12-21 in familial breast and ovarian cancer involve the wild-type chromosome. Nat. Genet. 2 (2), 128–131. 10.1038/ng1092-128 1303261

[B188] Society A. C. (2019). Breast cancer facts and figures 2019–2020. Atlanta, GA: American Cancer Society Inc.

[B189] Song Y. Zhu Z. An Y. Zhang W. Zhang H. Liu D. (2013). Selection of DNA aptamers against epithelial cell adhesion molecule for cancer cell imaging and circulating tumor cell capture. Anal. Chem. 85 (8), 4141–4149. 10.1021/ac400366b 23480100

[B190] Sonuç M. N. Sezgintürk M. K. (2014). Ultrasensitive electrochemical detection of cancer associated biomarker HER3 based on anti-HER3 biosensor. Talanta 120, 355–361. 10.1016/j.talanta.2013.11.090 24468382

[B191] Sorlie T. Tibshirani R. Parker J. Hastie T. Marron J. Nobel A. (2003). Repeated observation of breast tumor subtypes in independent gene expression data sets. Proc. Natl. Acad. Sci. USA 100 (14), 8418–8423. 10.1073/pnas.0932692100 12829800 PMC166244

[B192] Sparano J. A. Gray R. J. Makower D. F. Pritchard K. I. Albain K. S. Hayes D. F. (2015). Prospective validation of a 21-gene expression assay in breast cancer. N. Engl. J. Med. 373 (21), 2005–2014. 10.1056/NEJMoa1510764 26412349 PMC4701034

[B193] Streckfus C. F. Bigler L. R. Zwick M. (2006). The use of surface-enhanced laser desorption/ionization time-of-flight mass spectrometry to detect putative breast cancer markers in saliva: a feasibility study. J. Oral. Pathol. Med. 35 (5), 292–300. 10.1111/j.1600-0714.2006.00427.x 16630293

[B194] Sugimoto M. Wong D. T. Hirayama A. Soga T. Tomita M. (2010). Capillary electrophoresis mass spectrometry-based saliva metabolomics identified oral, breast and pancreatic cancer-specific profiles. Metabolomics 6 (1), 78–95. 10.1007/s11306-009-0178-y 20300169 PMC2818837

[B195] Tentler J. J. Tan A. C. Weekes C. D. Jimeno A. Leong S. Pitts T. M. (2012). Patient-derived tumour xenografts as models for oncology drug development. Nat. Rev. Clin. Oncol. 9 (6), 338–350. 10.1038/nrclinonc.2012.61 22508028 PMC3928688

[B196] Thompson D. Duedal S. Kirner J. McGuffog L. Last J. Reiman A. (2005). Cancer risks and mortality in heterozygous ATM mutation carriers. J. Natl. Cancer Inst. 97 (11), 813–822. 10.1093/jnci/dji141 15928302

[B197] Tian M. Cui Y. Z. Song G. H. Zong M. J. Zhou X. Y. Chen Y. (2008). Proteomic analysis identifies MMP-9, DJ-1 and A1BG as overexpressed proteins in pancreatic juice from pancreatic ductal adenocarcinoma patients. BMC cancer 8 (1), 241. 10.1186/1471-2407-8-241 18706098 PMC2528014

[B198] Tibshirani R. Hastie T. Narasimhan B. Chu G. (2002). Diagnosis of multiple cancer types by shrunken centroids of gene expression. Proc. Natl. Acad. Sci. USA 99 (10), 6567–6572. 10.1073/pnas.082099299 12011421 PMC124443

[B199] Torrente-Rodríguez R. M. Campuzano S. López-Hernández E. Montiel V. R.-V. Barderas R. Granados R. (2015). Simultaneous detection of two breast cancer-related miRNAs in tumor tissues using p19-based disposable amperometric magnetobiosensing platforms. Biosens. Bioelectron. 66, 385–391. 10.1016/j.bios.2014.11.047 25481114

[B200] Treilleux I. Arnedos M. Cropet C. Wang Q. Ferrero J. M. Abadie-Lacourtoisie S. (2015). Translational studies within the TAMRAD randomized GINECO trial: evidence for mTORC1 activation marker as a predictive factor for everolimus efficacy in advanced breast cancer. Ann. Oncol. 26 (1), 120–125. 10.1093/annonc/mdu497 25361980

[B201] Tsai M. Lo S. Audeh W. Qamar R. Budway R. Levine E. (2018). Association of 70-gene signature assay findings with physicians' treatment guidance for patients with early breast cancer classified as intermediate risk by the 21-gene assay. JAMA Oncol. 4 (1), e173470. 10.1001/jamaoncol.2017.3470 29075751 PMC5833645

[B202] Tuerk C. Gold L. (1990). Systematic evolution of ligands by exponential enrichment: RNA ligands to bacteriophage T4 DNA polymerase. Science 249 (4968), 505–510. 10.1126/science.2200121 2200121

[B203] Turner N. C. Reis-Filho J. S. (2006). Basal-like breast cancer and the BRCA1 phenotype. Oncogene 25 (43), 5846–5853. 10.1038/sj.onc.1209876 16998499

[B204] Van De Vijver M. J. He Y. D. Van’t Veer L. J. Dai H. Hart A. A. Voskuil D. W. (2002). A gene-expression signature as a predictor of survival in breast cancer. N. Engl. J. Med. 347 (25), 1999–2009. 10.1056/NEJMoa021967 12490681

[B205] van den Broek A. J. Schmidt M. K. van’t Veer L. J. Tollenaar R. A. van Leeuwen F. E. (2015). Worse breast cancer prognosis of BRCA1/BRCA2 mutation carriers: what’s the evidence? A systematic review with meta-analysis. PloS one 10 (3), e0120189. 10.1371/journal.pone.0120189 25816289 PMC4376645

[B206] Van Poznak C. Somerfield M. R. Bast R. C. Cristofanilli M. Goetz M. P. Gonzalez-Angulo A. M. (2015). Use of biomarkers to guide decisions on systemic therapy for women with metastatic breast cancer: American Society of Clinical Oncology Clinical Practice Guideline. J. Clin. Oncol. 33 (24), 2695. 10.1200/JCO.2015.61.1459 26195705 PMC5478102

[B207] van Reesema L. L. S. Zheleva V. Zheleva J. S. Jansen R. J. O’Connor C. F. Isbell A. J. (2016). SIAH and EGFR, two RAS pathway biomarkers, are highly prognostic in locally advanced and metastatic breast cancer. EBioMedicine 11, 183–198. 10.1016/j.ebiom.2016.08.014 27569656 PMC5049993

[B208] Van Sprundel T. Schmidt M. Rookus M. Brohet R. Van Asperen C. Rutgers E. J. (2005). Risk reduction of contralateral breast cancer and survival after contralateral prophylactic mastectomy in BRCA1 or BRCA2 mutation carriers. Br. J. Cancer 93 (3), 287–292. 10.1038/sj.bjc.6602703 16052221 PMC2361560

[B209] Varn F. S. Mullins D. W. Arias-Pulido H. Fiering S. Cheng C. (2017). Adaptive immunity programmes in breast cancer. Immunology 150 (1), 25–34. 10.1111/imm.12664 27564847 PMC5341497

[B210] Veeraraghavan J. De Angelis C. Reis-Filho J. S. Pascual T. Prat A. Rimawi M. F. (2017). De-escalation of treatment in HER2-positive breast cancer: determinants of response and mechanisms of resistance. Breast 34 Suppl 1, S19–S26. 10.1016/j.breast.2017.06.022 28687441 PMC6050048

[B211] Verhoog L. Brekelmans C. Seynaeve C. Van den Bosch L. Dahmen G. Van Geel A. (1998). Survival and tumour characteristics of breast-cancer patients with germline mutations of BRCA1. Lancet 351 (9099), 316–321. 10.1016/s0140-6736(97)07065-7 9652611

[B212] Wachter D. L. Hartmann A. Beckmann M. W. Fasching P. A. Hein A. Bayer C. M. (2014). Expression of neuroendocrine markers in different molecular subtypes of breast carcinoma, Biomed. Res. Int., 2014, 408459. 10.1155/2014/408459 24701575 PMC3950407

[B213] Walsh T. Casadei S. Lee M. K. Pennil C. C. Nord A. S. Thornton A. M. (2011). Mutations in 12 genes for inherited ovarian, fallopian tube, and peritoneal carcinoma identified by massively parallel sequencing. Proc. Natl. Acad. Sci. USA 108 (44), 18032–18037. 10.1073/pnas.1115052108 22006311 PMC3207658

[B214] Wang X. Yu H. Lu D. Zhang J. Deng W. (2014, Label free detection of the breast cancer biomarker CA15.3 using ZnO nanorods coated quartz crystal microbalance. Sensors Actuators B: Chem. 195, 630–634. 10.1016/j.snb.2014.01.027

[B215] Whittemore A. S. Gong G. John E. M. McGuire V. Li F. P. Ostrow K. L. (2004). Prevalence of BRCA1 mutation carriers among U.S. non-Hispanic Whites. Cancer Epidemiol. Biomarkers Prev. 13 (12), 2078–2083. 15598764

[B216] Won J. Y. Choi J.-W. Min J. (2013). Micro-fluidic chip platform for the characterization of breast cancer cells using aptamer-assisted immunohistochemistry. Biosens. Bioelectron. 40 (1), 161–166. 10.1016/j.bios.2012.07.004 22841444

[B217] Wu X. Xiao T. Luo Z. He R. Cao Y. Guo Z. (2018). A micro-/nano-chip and quantum dots-based 3D cytosensor for quantitative analysis of circulating tumor cells. J. Nanobiotechnology 16 (1), 1–9. 10.1186/s12951-018-0390-x 30205821 PMC6131777

[B218] Wurth R. Tarn K. Jernigan D. Fernandez S. V. Cristofanilli M. Fatatis A. (2015). A preclinical model of inflammatory breast cancer to study the involvement of CXCR4 and ACKR3 in the metastatic process. Transl Oncol. 8 (5), 358–367. 10.1016/j.tranon.2015.07.002 26500026 PMC4631055

[B219] Yang H. Yuan R. Chai Y. Mao L. Su H. Jiang W. (2011). Electrochemical immunosensor for detecting carcinoembryonic antigen using hollow Pt nanospheres-labeled multiple enzyme-linked antibodies as labels for signal amplification. Biochem. Eng. J. 56 (3), 116–124. 10.1016/j.bej.2011.04.004

[B220] Yousef E. M. Tahir M. R. St-Pierre Y. Gaboury L. A. (2014). MMP-9 expression varies according to molecular subtypes of breast cancer. BMC cancer 14 (1), 609–612. 10.1186/1471-2407-14-609 25151367 PMC4150970

[B221] Zhang H. Y. Liang F. Jia Z. L. Song S. T. Jiang Z. F. (2013). mutation, methylation and expression in breast cancer patients. Oncol. Lett. 6 (1), 161–168. 10.3892/ol.2013.1331 23946797 PMC3742525

[B222] Zhang J. Wu D. Z. Cai S. X. Chen M. Xia Y. K. Wu F. (2016). An immobilization-free electrochemical impedance biosensor based on duplex-specific nuclease assisted target recycling for amplified detection of microRNA. Biosens. Bioelectron. 75, 452–457. 10.1016/j.bios.2015.09.006 26363493

[B223] Zhang L. Ridgway L. D. Wetzel M. D. Ngo J. Yin W. Kumar D. (2013). The identification and characterization of breast cancer CTCs competent for brain metastasis. Sci. Transl. Med. 5 (180), 180ra48. 10.1126/scitranslmed.3005109 PMC3863909 23576814

[B224] Zhang L. Xiao H. Karlan S. Zhou H. Gross J. Elashoff D. (2010). Discovery and preclinical validation of salivary transcriptomic and proteomic biomarkers for the non-invasive detection of breast cancer. PloS one 5 (12), e15573. 10.1371/journal.pone.0015573 21217834 PMC3013113

[B225] Zhang Y. Schnabel C. A. Schroeder B. E. Jerevall P. L. Jankowitz R. C. Fornander T. (2013). Breast cancer index identifies early-stage estrogen receptor-positive breast cancer patients at risk for early- and late-distant recurrence. Clin. Cancer Res. 19 (15), 4196–4205. 10.1158/1078-0432.CCR-13-0804 23757354

[B226] Zhao J. Wu J. Cai H. Wang D. Yu L. Zhang W.-H. (2016). E3 Ubiquitin ligase Siah-1 is down-regulated and fails to target natural HBx truncates for degradation in hepatocellular carcinoma. J. Cancer 7 (4), 418. 10.1158/1078-0432.CCR-13-0804 26918055 PMC4749362

[B227] Zhao S. Yang W. Lai R. Y. (2011). A folding-based electrochemical aptasensor for detection of vascular endothelial growth factor in human whole blood. Biosens. Bioelectron. 26 (5), 2442–2447. 10.1016/j.bios.2010.10.029 21081271

[B228] Zhou J. Liu T. Wang W. (2018). Prognostic significance of matrix metalloproteinase 9 expression in osteosarcoma: a meta-analysis of 16 studies. Medicine 97 (44), e13051. 10.1097/md.0000000000013051 30383677 PMC6221749

